# Histopathological studies of nonhuman primates exposed to supralethal doses of total- or partial-body radiation: influence of a medical countermeasure, gamma-tocotrienol

**DOI:** 10.1038/s41598-024-56135-w

**Published:** 2024-03-08

**Authors:** Vijay K. Singh, Stephen Y. Wise, Oluseyi O. Fatanmi, Sarah A. Petrus, Alana D. Carpenter, Sang-Ho Lee, Martin Hauer-Jensen, Thomas M. Seed

**Affiliations:** 1 Division of Radioprotectants, Department of Pharmacology and Molecular Therapeutics, F. Edward Hébert School of Medicine, Uniformed Services University of the Health Sciences, 4301 Jones Bridge Road, Bethesda, MD 20814-2712 USA; 2 Armed Forces Radiobiology Research Institute, Uniformed Services University of the Health Sciences, Bethesda, MD 20814 USA; 3 Pathology Department, Research Services, Naval Medical Research Center, Silver Spring, MD 20910 USA; 4 Division of Radiation Health, Department of Pharmaceutical Sciences, University of Arkansas for Medical Sciences, Little Rock, AR 72205 USA; 5 Tech Micro Services, 4417 Maple Avenue, Bethesda, MD 20814 USA

**Keywords:** Supralethal lethal radiation, Histopathology, Nonhuman primates, Total- partial-body irradiation, Organ system injury, Cell biology, Cell death

## Abstract

Despite remarkable scientific progress over the past six decades within the medical arts and in radiobiology in general, limited radiation medical countermeasures (MCMs) have been approved by the United States Food and Drug Administration for the acute radiation syndrome (ARS). Additional effort is needed to develop large animal models for improving the prediction of clinical safety and effectiveness of MCMs for acute and delayed effects of radiation in humans. Nonhuman primates (NHPs) are considered the animal models that reproduce the most appropriate representation of human disease and are considered the gold standard for drug development and regulatory approval. The clinical and histopathological effects of supralethal, total- or partial-body irradiations (12 Gy) of NHPs were assessed, along with possible protective actions of a promising radiation MCM, gamma-tocotrienol (GT3). Results show that these supralethal radiation exposures induce severe injuries that manifest both clinically as well as pathologically, as evidenced by the noted functionally crippling lesions within various major organ systems of experimental NHPs. The MCM, GT3, has limited radioprotective efficacy against such supralethal radiation doses.

## Introduction

There are significant health risks associated with ionizing radiation (IR) exposures that arise from accidental, military or terrorist-driven radiological/nuclear events^1^. The origin and nature of such unwanted exposures and their related health consequences vary widely, but at the extremes, these noted health effects range from acute and immediately life-threatening to subacute or chronic and very delayed in terms of presentation^2^. The intensity, duration and quality of these IR exposures are the principal biophysical drivers of such health effects and are primary determinants of the incidence, type, and severity of those injuries, i.e., adverse health effects^3^.

At the extremes of the exposure continuum, low doses or intensities of exposure tend to accentuate the stochastic risks to a number of prominent, late-arising disease entities, such as cancer, whereas extremely high doses or intensities of irradiation promote deterministically early arising diseases, such as the acute radiation syndrome that may substantially increase both mortality and morbidity within fractions of the at risk exposed population^3^. Full knowledge of the nature of these types of radiation exposure related injuries/diseases is essential in order to develop and to implement medical contingency plans for such radiological/nuclear exposure events.

Relative to the current status of available medical countermeasures (MCMs) for the ARS, there are only six medicinals currently approved by the United States Food and Drug Administration (US FDA) and all six are medically indicated to treat the hematopoietic component of ARS (H-ARS). These six agents and their specific indications include Neupogen, Neulasta (PEGfilgrastim), Udenyca (PEGfilgrastim-cbqv), Stimufend (PEGfilgrastim-fpgk) and Leukine, which are approved to treat H-ARS-associated neutropenia, while Nplate is approved to treat H-ARS-associated thrombocytopenia^4–16^. Currently, there are no FDA-approved MCMs for either the gastrointestinal sub-syndrome of ARS (GI-ARS) or for ARS’s neurovascular component (NV-ARS), both of which develop following significantly high doses of radiation^2^.

While the pathological consequences and ‘recovery’ processes (or lack thereof) are reasonably well understood and documented in primates subjected to low or moderate levels of IR (e.g., sub-lethal doses of 1–3 Gy) to higher, mid-lethal exposure levels (e.g., 5–8 Gy), pathological processes associated with extremely high, supralethal exposures (e.g., NVS eliciting IR exposures) are not as well documented and are certainly less well understood in terms of specific pathogenic processes with select tissues and organ systems of the body^17–21^. Similarly, these uncertainties that relate to the pathological processes under extremely high IR doses extend to possible recovery processes of tissue/organ systems that might be initiated and/or augmented following select MCM intervention(s). As such, these ‘baseline’ pathological studies of supralethal IR exposures are deemed essential for the development of new and promising MCMs under study.

MCM development under the US FDA Animal Rule requires validated animal models of radiation injury^22–25^. Several types of radiation exposures including total- and partial-body as well as whole thorax lung irradiation (TBI, PBI and WTLI) have been used for understanding radiation injury, developing MCMs, and identifying biomarkers. The WTLI approach focuses on specific organ sequelae and the relationship of such exposure parameters to those of a radiation/nuclear scenario that may be marginal due to the uniform organ-specific exposure, which is unlikely under any radiation/nuclear event^26^. Significant knowledge gaps exist in model development relative to various types of radiation exposures. The progression of multi-organ injury during ARS requires additional model development using high-dose TBI and PBI as well as in-depth study of radiation-induced injury within various organs.

The NHP has been a valuable large animal model for studying various sub-syndromes of ARS since these animals are closest to humans in respect of pathophysiology and genetic homology^27–29^. There is more than 95% homology between the DNA sequence of the genus Macaca and humans. Such a close relationship between macaques and humans has made these animals attractive as animal models for biomedical research, specifically for drug development and for the identification of biomarkers^23,30^. The NHP model closely recapitulates the clinical and histopathological characteristics of radiation induced injuries of comparably irradiated humans^27^. Additionally, due to the extended life span and similar supportive care requirements for ARS treatment in NHPs and humans, it is easy to compare the dose effect relationships.

Gamma-tocotrienol (GT3; a member of vitamin E family) has been extensively evaluated for its radioprotective efficacy in both rodents (mice) and NHP models^31,32^ and this agent has been selected for advanced development as a radiation MCM^32–34^. Studies have demonstrated that GT3 has significant levels of radioprotectiveness for both H-ARS and GI-ARS when tested in a murine model of acute radiation injury^31,32,35,36^. GT3 has also been shown to induce high levels of granulocyte colony-stimulating factor (G-CSF) and mobilize progenitors, and it enhances the radioprotective efficacy of low doses of amifostine (another important radioprotector)^35,37–39^. These initial findings prompted us to initiate advanced, large animal studies using NHPs^34,40–45^. We have also conducted studies using various omics platforms to identify its biomarkers which will be helpful to obtain regulatory approval following US FDA Animal Rule^44–51^. With additional investigation, this agent may prove to be highly useful and have safe prophylaxis for potentially lethal effects of acute radiation injuries. It is important to note that a single injection of GT3 without any supportive care was equivalent, in terms of improving hematopoietic recovery, to multiple doses of Neupogen and two doses of Neulasta with full supportive care (including blood products) in the NHP model^7,11^. GT3 may serve as an ultimate radioprotector for use in humans, particularly for military personnel and first responders. Current research and development of this agent and its positive findings of safety and efficacy profiles seem to be setting the stage for possible approval by the US FDA for human use in the future.

**Figure 1:**
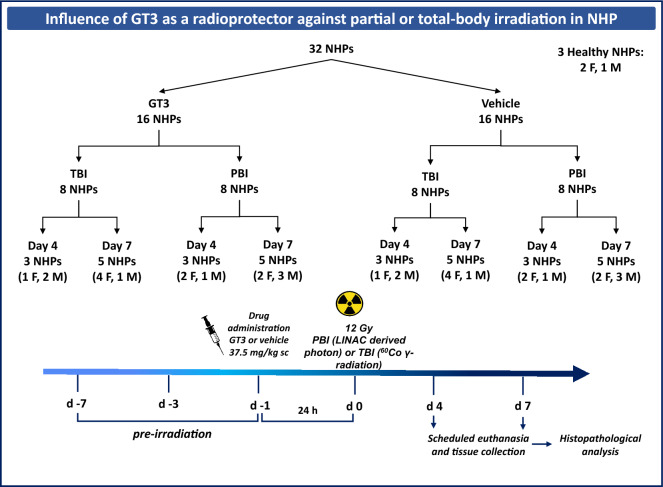
Experimental design for total- and partial-body irradiation and tissue collection for histopathology. Sixteen NHPs were pretreated with GT3 or vehicle and then irradiated. Animals were euthanized on day 4 or day 7 to collect various tissues for histopathology. *NHP* Nonhuman primate, *GT3* Gamma-tocotrienol, *TBI* Total-body irradiation, *PBI* Partial-body irradiation, *F* Female, *M* Male.

In this study, we have used NHPs, a highly translational model system, to study the histopathology of various organs from animals exposed to total- and partial-body supralethal doses of 12 Gy. Cobalt-60 gamma-radiation and linear accelerator-derived photon were used for TBI and PBI, respectively (Fig. 1). Our results demonstrate that GT3 provides minimal protection to various tissue types at supralethal doses of either TBI or PBI.

## Results

### Clinical status

The clinical condition of all animals under test were monitored and recorded continuously throughout the testing period. Primary metrics of clinical status are described with major findings listed. In brief, body weight (Table 1, Supplementary Table 2), body temperature (Table 2, Supplementary Table 3), heart rate (Table 3, Supplementary Table 4), blood pressure (Table 4, Supplementary Table 5), gross pathology (Table 5), and histopathology (Table 6) of all supralethally irradiated animals declined relative to pre-exposure values (or relative to unirradiated control animals) and some markedly so, during the week following IR exposures. Vital signs data is illustrated in Supplementary Fig. 1.

**Table 1:** Body weights of NHPs subjected to supralethal IR exposures (TBI or PBI) and treated with GT3 or vehicle.

Radiation	Treatment	Euthanasia (day)	Age (years)	(N size)—Average ± SD		
				Total	Males	Females
PBI	Vehicle	4	3.8 ± 0.2	(3)—6.33 ± 0.58	(1)—6.00	(2)—6.50 ± 0.71
		7	3.8 ± 0.1	(5)—10.80 ± 1.92	(3)—11.3 ± 1.53	(2)—10.00 ± 2.80
	GT3	4	3.8 ± 0.2	(3)—3.00 ± 2.65	(1)—6.00	(2)—1.50 ± 0.71
		7	3.8 ± 0.1	(5)—12.20 ± 2.28	(3)—11.00 ± 1.00	(2)—14.00 ± 2.83
TBI	Vehicle	4	4.5 ± 0.7	(3)—6.33 ± 2.52	(2)—6.50 ± 3.53	(1)—6.00
		7	4.2 ± 0.5	(5)—10.8 ± 2.77	(1)—8.00	(4)—11.50 ± 2.65
	GT3	4	4.1 ± 0.9	(3)—0.67 ± 2.08	(2)—1.00 ± 2.83	(1)—0.00
		7	4.7 ± 0.7	(5)—12.20 ± 4.02	(1)—8.00	(4)—13.25 ± 3.77

*SD* standard deviation.

**Table 2:** Body temperatures of NHPs subjected to supralethal IR exposures (TBI or PBI) and treated with GT3 or vehicle.

Radiation	Treatment	Euthanasia (day)	Temperature (Celsius) recorded post-irradiation (day)		
			Average ± SD for day -3	Average ± SD for day 4	Average ± SD for day 7
PBI	Vehicle	4	39.50 ± 0.15	38.76 ± 0.32	
		7	39.72 ± 0.09	39.20 ± 0.67	36.66 ± 1.57
	GT3	4	38.04 ± 0.70	37.98 ± 1.30	
		7	39.54 ± 0.27	38.93 ± 0.68	35.43 ± 1.23
TBI	Vehicle	4	38.83 ± 0.44	38.22 ± 0.00	
		7	38.19 ± 0.43	38.58 ± 0.51	36.79 ± 0.77
	GT3	4	37.91 ± 1.27	38.48 ± 0.50	
		7	37.91 ± 0.40	38.27 ± 0.34	37.46 ± 1.75

*SD* standard deviation.

**Table 3:** Heart rates of NHPs subjected to supralethal IR exposures (TBI or PBI) and treated with GT3 or vehicle.

Radiation	Treatment	Euthanasia (day)	Heart Rate (BPM) recorded post-irradiation (day)		
			Average ± SD for day -3	Average ± SD for day 4	Average ± SD for day 7
PBI	Vehicle	4	171 ± 22	210 ± 6	
		7	233 ± 22	225 ± 12	178 ± 33
	GT3	4	190 ± 45	208 ± 25	
		7	200 ± 19	213 ± 19	140 ± 27
TBI	Vehicle	4	249 ± 23	160 ± 24	
		7	236 ± 33	228 ± 25	173 ± 13
	GT3	4	159 ± 39	160 ± 7	
		7	202 ± 61	223 ± 27	187 ± 17

*SD* standard deviation.

**Table 4:** Blood pressures of NHPs subjected to supralethal IR exposures (TBI or PBI) and treated with GT3 or vehicle.

Radiation	Treatment	Euthanasia (day)	Blood pressure recorded post-irradiation (day)		
			Average ± SD for day -3	Average ± SD for day 4	Average ± SD for day 7
PBI	Vehicle	4	146/93 ± 24/36	126/80 ± 24/12	
		7	120/74 ± 24/20	129/88 ± 16/14	117/89 ± 21/18
	GT3	4	132/98 ± 27/26	124/76 ± 16/25	
		7	148/97 ± 11/10	121/77 ± 16/12	118/80 ± 16/20
TBI	Vehicle	4	160/93 ± 15/21	118/61 ± 10/24	
		7	168/98 ± 20/15	159/91 ± 20/29	116/78 ± 19/13
	GT3	4	136/73 ± 24/12	102/59 ± 6/9	
		7	137/83 ± 36/25	153/93 ± 11/23	128/89 ± 31/26

*SD* standard deviation.

**Table 5:** Gross pathological findings of NHP tissues from animals with TBI or PBI and treated with GT3 or vehicle.

Radiation	Treatment	Euthanasia (day)	NHP#	Sex	Age (years)	Gross pathological findings
PBI	Vehicle	4	1608024	F	3.5	Overall, a supralethal dose of radiation caused significant injury to the organ system, inflammatory response manifested in the lung, animal observed to be 10% dehydrated, moderate generalized injected vasculature of the lung tissue, multifocal, raised lesions along the dorsal surface of the lungs, no redness associated with lesions observed
			1603151	M	3.9	Inflammatory response manifested in the lung, both lungs observed to have few lesions, but no redness, animals appeared to be euhydrated, all other organs and tissues found to be within normal limits of size, shape, and consistency
			1603060	F	3.9	A few lesions noted in both lungs, animal appeared to be euhydrated, moderate multifocal hemorrhagic congestion in the right middle lung lobe
		7	1607024	F	3.6	Inflammatory response manifested in the lung, dehydration consistent with > 12 – 15% weight loss, mild to moderate generalized injected vasculature concentrated at the dorsal surface of the lung tissue, microsplenia (shriveled spleen), stomach full of straw-colored liquid content with petechial hemorrhage on the gastric walls noticed
			1604091	M	3.8	Inflammatory response manifested in the lung, dehydration consistent with > 12 – 15% weight loss, several ulcerative lesions noted in both lungs, copious amount of pericardial fluid present around the heart, bruising and hemorrhages noted all over body, GI tract presented with significant petechiae in several regions
			1605536	F	3.8	Dehydration consistent with > 15% weight loss, animal presented with sunken eyes, pale skin and dry mucus membranes, shriveled heart due to possible decreased pericardial fluid
			1603103	M	3.9	Inflammatory response manifested in the lung, dehydration consistent with > 10 – 15% weight loss, pale skin, bruising, and dry mucus membranes, moderate generalized hemorrhagic congestion throughout the lung tissue, focal, pale lesion present on the surface of the liver, gas-distended stomach, petechiae and hemorrhage noted throughout GI tract
			1603155	M	3.9	Gastrointestinal findings consistent with poor appetite, focal liver congestion noted to be a postmortem change, dehydration consistent with > 10 – 15% weight loss, animal presented with sunken eyes, pale skin, bruising, non-healing wounds, and dry mucus membranes, stomach empty, shriveled and few intussusceptions noted in the small intestines, all other organs and tissues found to be within normal limits of size, shape, and consistency
	GT3	4	1603080	F	3.9	Inflammatory response manifested in the lung and spleen, animal observed to be euhydrated, few generalized injected vasculature of the lung tissue, roughened edges of the splenic surface presented with dark color
			1608009	F	3.5	Severe hemorrhagic congestion of the lungs, right cranial, middle, accessory lung lobes have moderate to severe hemorrhagic congestion, left cranial and caudal lung lobes have mild to moderate multifocal hemorrhagic congestion, all other organs and tissues found to be within normal limits of size, shape, and consistency, animal observed to be euhydrated
			1603051	M	3.9	Inflammatory response manifested in the lung, dehydration consistent with > 10% weight loss, few lesions noted on both lungs with petechiae and hemorrhage
		7	1606094	F	3.7	Inflammatory response manifested in the lung, mild to moderate lesions noted in both lungs, heart contained decreased pericardial fluid, few raised projections on the liver surface, shriveled spleen, GI tract presented with several petechiae and hemorrhage, and mild to moderate gas distension in the stomach, dehydration consistent with > 10—12% weight loss
			1604041	M	3.8	Inflammatory response manifested in the lung, moderate generalized injected vasculature of the lung tissue, few lesions noted in upper and middle lobes of right lung, rough edges of the spleen, left kidney firm to touch, animal observed to be euhydrated
			1603158	F	3.9	Dehydration consistent with > 10 – 15% weight loss, animal presented with sunken eyes, pale skin and mucus membranes, hyperinflation throughout the lung tissue with few lesions noted on both lungs
			1603047	M	3.9	Poor appetite, moderate gas distended stomach and intussusception noted in small intestines, all other organs and tissues found to be within normal limits of size, shape, and consistency, dehydration consistent with > 10 – 15% weight loss
			1606085	M	3.7	Both lungs pale and hyperinflated, and few lesions noted in right lung, gastrointestinal findings consistent with poor appetite, empty stomach, dry intestinal tract, and mucus membranes, dehydration consistent with > 20% weight loss, all other organs and tissues found to be within normal limits of size, shape, and consistency
TBI	Vehicle	4	RA2619	F	5.0	Moderate redness noted in both lungs, roughened edges on the splenic surface, all other organs and tissues found to be within normal limits of size, shape, and consistency
			RA2692	M	4.9	A few minute red specks noted on both lungs, all other organs and tissues found to be within normal limits of size, shape, and consistency
			RA2892	M	3.7	All organs and tissues found to be within normal limits of size, shape, and consistency, obvious exposure to supralethal irradiation
		7	RA2378	F	5.0	Severe hemorrhagic congestion of both lungs, few petechiae noted in jejunum and colon, roughened edges on the splenic surface, enlarged spleen, a couple of intussusceptions noted in the small intestines, petechiae and hemorrhage observed in lining of small intestines, dehydration consistent with > 10% weight loss
			RA2931	M	4.2	Significant GI damage present with hemorrhage, petechiae, moderate bruising all over body, a few non-healing wounds, a few skin ulcers, gas noted in the stomach, mild dehydration, watery stool in large intestine, dehydration consistent with body weight loss
			RA2922	F	4.2	Redness and bruising noted all over body, many skin ulcers possibly from non-healing wounds, moderate petechiae noted in both lungs, roughened surfaces of spleen and kidneys, severe hemorrhage and petechiae in entire GI tract, dehydration consistent with > 10% weight loss
			RA3238	F	3.8	Several lesions noted on both lungs, pale kidneys, inflamed gall bladder with foul smelling, grainy bile, petechiae and hemorrhage noted in regions of the small intestines and colon; few blood clots noted in the stomach, shriveled bladder, dehydration consistent with > 10% weight loss
			RA3291	F	3.9	Bruising and mild to moderate petechiae noted all over body, colon contained red tarry stools, a couple of intussusceptions noted in the small intestines, inflamed gallbladder with grainy, dark green bile, moderate dehydration noted in abdominal cavity, ulcerative lesions noted in the lungs, spleen, and kidneys
	GT3	4	RA2599	M	3.7	All other organs and tissues found to be within normal limits of size, shape, and consistency, obvious sign of supralethal radiation exposure
			RA2781	M	5.1	Minimal redness noted in both lungs, all other organs and tissues found to be within normal limits of size, shape, and consistency
			RA2115	F	5.5	All other organs and tissues found to be within normal limits of size, shape, and consistency, dehydrated
		7	RA2829	M	5.2	Moderate GI damage present with few hemorrhages, petechiae, mild bruising in some parts of the body, mild dehydration, red–black, tarry stools in large intestine, intussusception in colon
			RA2626	F	5.2	Moderate hemorrhage noted inside stomach, up to duodenal junction, ileocecal junction contained mild to moderate hemorrhage, petechiae noted in entire GI tract, moderate dehydration noted throughout GI tissue, dehydration consistent with > 10% weight loss
			RA2528	F	4.0	Several lesions noted on heart surface, both lungs, pale kidneys, slightly shriveled spleen, petechiae and hemorrhage noted in regions of the small intestines and colon; dehydration consistent with > 10% weight loss
			RA3246	F	3.9	Notable lesions in the upper and middle portions of both lungs, petechiae and hemorrhage noted in regions of the small intestines and colon; dehydration consistent with > 10% weight loss
			RA2762	F	5.0	Significant GI tract damage, lesions noted in lungs, notable petechiae and hemorrhage in the entire GI tract, intussusception in small regions of the small intestine, gas noted in the upper GI, mild dehydration, redness on the surface on the intestines, shriveled bladder, dehydration consistent with > 10% weight loss

**Table 6:** Histopathological scoring of various organs of healthy animals in addition to TBI and PBI animals treated with GT3 or vehicle.

Organs	Average histopathology scores								
	TBI				PBI				AVG (range) Healthy (n = 3)
	Vehicle		GT3		Vehicle		GT3		
	AVG (range) day 4 (n = 3)	AVG (range) day 7 (n = 5)	AVG (range) day 4 (n = 3)	AVG (range) day 7 (n = 5)	AVG (range) day 4 (n = 3)	AVG (range) day 7 (n = 5)	AVG (range) day 4 (n = 3)	AVG (range) day 7 (n = 5)	
Sternum									
Cellular depletion	5	5	5	5	5	5	5	5	0
Spleen									
White pulp depletion	3	5 (4–5)	3 (3–4)	4 (4–5)	3 (3–4)	5 (4–5)	3 (3–4)	4 (3–5)	1 (0–2)
Duodenum									
Villi/crypt ratio	4 (3.8–4.6)	2 (1.4–3.5)	3 (2–4.2)	2 (1.3–1.7)	3 (1.6–4.8)	1 (0.9–1.8)	3 (1.9–4.8)	2 (1–2.1)	5 (4.5–5.9)
Inflammatory cell infiltrates	2 (1–2)	2 (1–3)	3 (2–3)	1 (1–2)	2 (1–3)	1 (1–2)	1 (1–2)	2	1 (0–1)
Crypt dilation	2 (1–2)	2 (1–3)	2	2 (2–3)	1 (1–2)	2 (1–3)	1 (0–2)	2 (1–3)	0
Villi fusion	2	2 (1–3)	2 (1–2)	2 (2–3)	2 (0–4)	3 (2–4)	1 (1–2)	3 (3–4)	0
Villi loss	2	3 (2–4)	2	3 (2–3)	2 (1–3)	3	1 (1–2)	3 (3–4)	0
Jejunum									
Villi/crypt ratio	4 (3.8–4.6)	2 (2.1–2.9)	6 (4.2–6.6)	4 (2.9–5)	7 (4.2–11)	3 (2.3–3.9)	7 (6.4–7.2)	2 (1.1–2.5)	6 (5.2–7)
Inflammatory cell infiltrates	2 (1–2)	1 (1–2)	1 (0–1)	1 (1–2)	1 (0–2)	1 (1–2)	1 (0–2)	2 (1–3)	1 (0–2)
Crypt dilation	1 (0–2)	2 (1–2)	1 (0–2)	1 (1–2)	1 (0 –2)	2 (0–3)	1	2 (1–4)	0
Villi fusion	1	1 (0–2)	1 (0–1)	1 (1–2)	1	2 (1–4)	1 (1–2)	4 (2–5)	0
Villi loss	2	2 (1–3)	1 (1–2)	2 (2–3)	2 (1–2)	2 (1–4)	2 (1–2)	4 (3–4)	0
Ileum									
Villi/crypt ratio	5 (3.1–6.2)	2 (1.5–2.5)	4 (3–4.4)	2 (1.2–2.2)	5 (4–7.7)	1 (0.8–2.1)	6 (4.6–6)	1 (0.5–0.9)	4 (3.8–4.9)
Inflammatory cell infiltrates	1 (1–2)	1 (1–2)	1 (0–2)	1 (0–3)	1	2 (1–2)	0 (0–1)	2 (1–2)	0
Crypt dilation	1 (0–1)	3 (2–3)	0	2 (1–3)	1 (0–1)	4 (3–4)	1 (0–1)	3 (2–4)	0
Villi fusion	1 (0–1)	1 (1–2)	2 (1–2)	2 (1–3)	1 (0–2)	3 (2–4)	0 (0–1)	4 (3–5)	1 (0–1)
Villi loss	2	3 (2–3)	2 (2–3)	3 (3–4)	2 (1–3)	3 (3–4)	2 (1–2)	4 (3–4)	1 (0–2)
Large intestine									
Inflammatory cell infiltrates	2 (1–3)	3 (2–3)	2	2 (2–3)	2	1 (1–2)	2 (1–2)	2 (1–2)	1 (0–2)
Crypt dilation	3 (1–4)	3 (3–4)	2 (1–3)	3 (3–4)	2 (1–4)	3 (3–4)	4	4 (3–4)	0
Crypt loss	2 (2–3)	4	1	4 (3–4)	2 (2–3)	3 (3–4)	2 (2–3)	4 (3–4)	0
Kidney									
Tubular degeneration	4	3	4	3 (2–4)	3 (3–4)	3 (2–4)	2 (2–3)	3 (2–4)	3 (2–3)
Tubular regeneration	0 (0–1)	0 (0–1)	0 (0–1)	1 (0–2)	1 (0–2)	2 (2–3)	1 (0–2)	2 (2–3)	1 (0–2)
Lung									
Alveolar septal degeneration	2 (2–3)	3 (3–4)	2 (1–3)	3 (2–3)	2 (1–3)	2 (1–2)	2 (2–3)	1 (0–3)	1 (0–3)
Alveolar edema	0	2 (1–3)	0	2 (0–3)	0	2 (0–3)	3 (1–4)	1 (0–4)	1 (0–3)
Septal cellularity	1	1 (0–1)	1	1 (0–1)	1	1	1	0 (0–1)	0 (0–1)
Alveolar emphysema	1	1 (0–2)	1 (0–1)	1 (0–2)	0	1 (0–1)	0	1 (0–1)	0 (0–1)

The number of animals per group assigned to a given score has been provided. For each score, there are 3 animals on day 4, 5 animals on day 7, and 3 healthy animals. AVG, average; n for each group is given below treatment. The severity of tissue damage was graded based on a point system, with 0 = none, 1 = minimal, 2 = mild, 3 = moderate, 4 = marked, and 5 = severe. It is important to note that villi/crypt values are ratios, and therefore were not graded on the 1–5 severity scale as was used for the other parameters. The following scale can be used to interpret villi/crypt ratios: < 2 = severe, 2.1–3 = marked, 3.1–4 = moderate, 4.1–5 = mild, 5.1–6 = minimal, and > 6.1 = none. For averages that do not include ranges, all animals in this group were scored the same.

### TBI-induced injuries in various organs

Organ-associated clinical and related histopathologic responses within supralethally irradiated NHPs (12 Gy TBI) were evaluated, with major findings described below. These evaluations of various tissues from select organ systems included: blood-forming/lymphohematopoietic tissues (sternum and spleen); GI tissues (small and large intestines: duodenum, jejunum, ileum, colon); liver; urogenital tissues (kidney and bladder); pulmonary (lung); and cardiovascular (heart) tissues. Average histopathological scores and standard deviations for each tissue collected from each group are listed in Table 6. For comparison, the scoring of tissue sections from a select number of healthy, unirradiated control animals are shown. It is important to note that the healthy animals do display some degree of abnormality in a few of the tissues analyzed. However, this is not uncommon for NHPs. There are many environmental factors including diet, stress, etc. that have an effect on the overall health of an animal. Considering that NHPs are obtained from different facilities around the world and are not bred and raised under the same conditions, it is logical to assume that there will be minor variances from animal to animal, and that these differences do not necessarily indicate that an animal is otherwise unhealthy. Other factors including age could also affect the histopathological scoring of these healthy animals.

#### Lymphohematopoietic tissues- clinical features and histopathology

**Figure 2:**
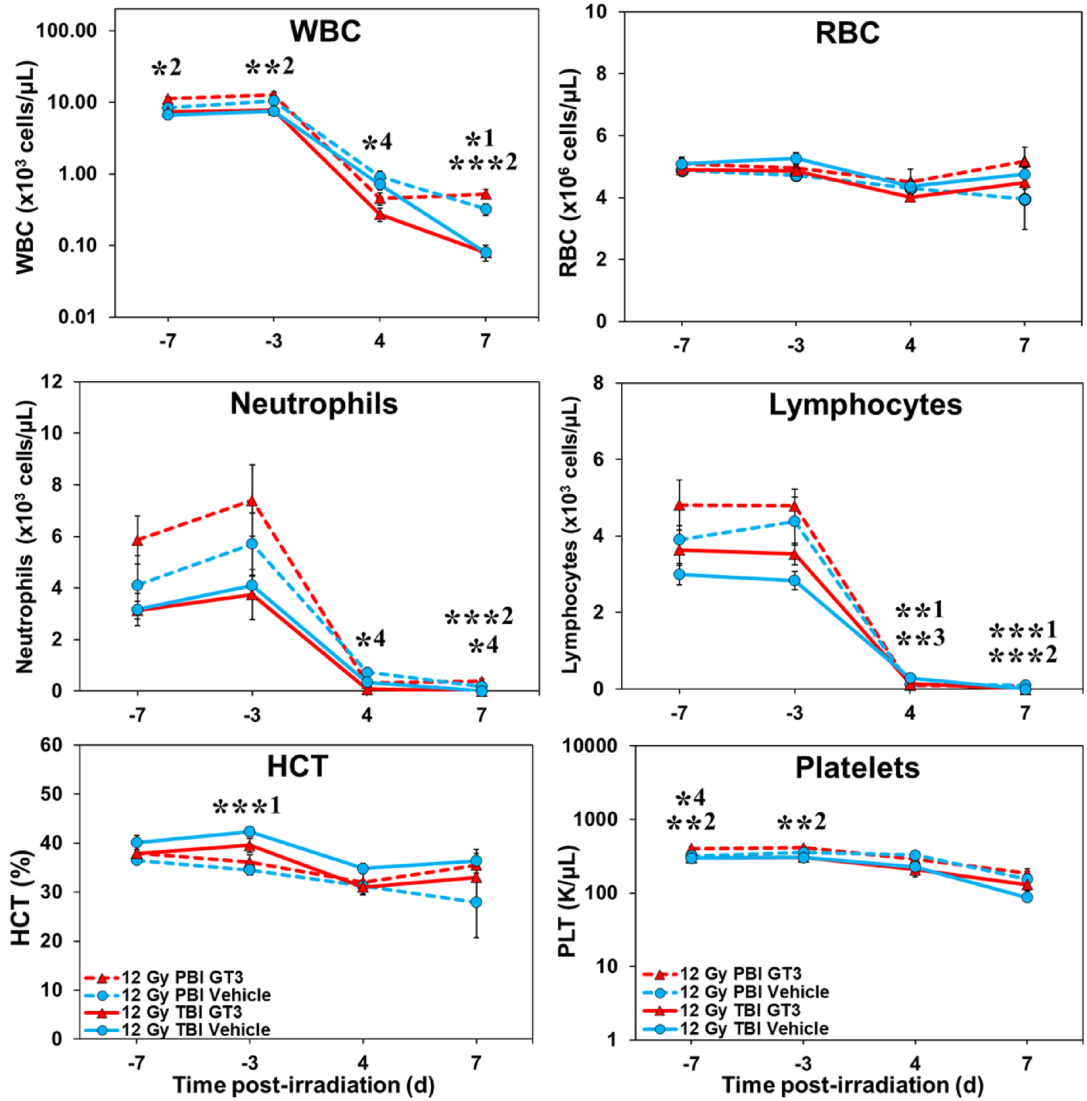
Effects of GT3 on white blood cells (WBCs), red blood cells (RBCs), neutrophils, lymphocytes, hematocrit (HCT%), and platelets in NHPs exposed to 12 Gy partial or total-body radiation. One-Way ANOVA with Tukey post-hoc tests were performed to determine significant differences between the following groups: TBI Vehicle vs PBI Vehicle (p < 0.05 *1, p < 0.01 **1, p < 0.001 ***1), TBI GT3 vs PBI GT3 (p < 0.05 *2, p < 0.01 **2, p < 0.001 ***2), TBI Vehicle vs TBI GT3 (p < 0.01 **3), and PBI Vehicle vs PBI GT3 (p < 0.05 *4).

Blood cell counts were altered regardless of exposure protocol (TBI or PBI). Circulating levels of blood leukocytes and platelets declined sharply (compared to pre-exposure levels) (Fig. 2). Red blood cell counts and related hematocrits showed a more gradual decline.

**Figure 3:**
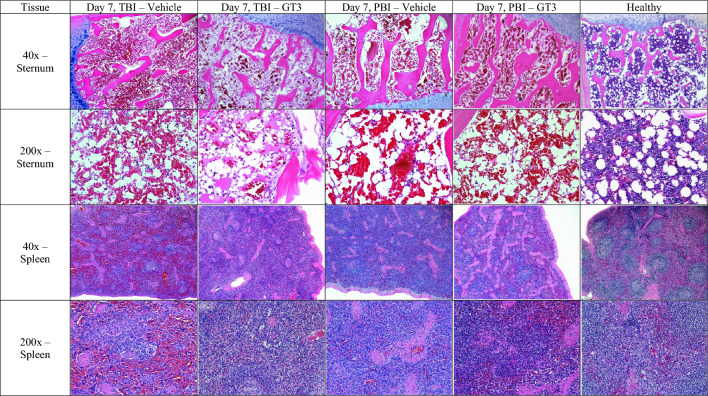
Comparison of lymphohematopoietic organs collected from vehicle- and GT3-treated NHPs day 7 post-irradiation with 12 Gy. Radiation exposure was either partial-body (PBI) or total-body (TBI). GT3 Sternum: TBI RA2528F, PBI 1603158F, Vehicle Sternum: TBI RA3238F, PBI 1607024F, Healthy 14041532F. GT3 Spleen: TBI RA2528F, PBI 1603158F, Vehicle Spleen: TBI RA3291F, PBI 1603103M, Healthy 14041532F.

These early blood-based clinical responses corresponded generally to the dramatic histopathological features of sternum and spleen samples taken at the time of euthanasia (Fig. 3, Supplementary Fig. 2, Table 6). All sternum sections, regardless of treatment or time point, showed pronounced cellular depletion (severe hypoplasia), with extensive loss of primary hematopoietic elements (Fig. 3, Supplementary Fig. 2, Table 6). Marrow cavities were partially filled by adipocytes, along with moderate levels of hemorrhage for the TBI animals. In contrast, there were abundant hematopoietic cells and precursors expanding the marrow space in unirradiated control animals (Fig. 3, Supplementary Fig. 2).

Spleen sections collected on day 4 showed either minimal or no difference in the loss of white pulp in both treatment groups (Fig. 3, Supplementary Fig. 2). However, for the spleen sections collected on day 7 regardless of treatment, diffuse to severe loss of white pulp were noted both within the inner germinal zone and the outer mantle and peripheral zones (Fig. 3, Supplementary Fig. 2). On day 7, there was marked to severe white pulp loss for both treatment groups, but the vehicle-treated group was characterized by more frequent and severe loss than those of the GT3-treated group (Table 6).

However, there were well-defined histopathological differences between day 4 and day 7 for both treatment groups (Fig. 3). The extent of white pulp loss was noticeably greater for the day 7 sections for both vehicle- and GT3-treated groups. These differences reflect the time progression of lymphocyte loss from lesser destruction of lymphocytes in day 4 to increased overall destruction of lymphocytes by day 7.

#### GI tissues- clinical features and histopathology

Mild to moderate dehydration was a common occurrence noted in the animals. In addition, animals displayed lethargy and malaise, inappetence, and a hunched posture during the course of the study. The presence of bloody stools and uncontrolled defecations reflected severe GI disturbances.

##### Small intestine/duodenum

**Figure 4:**
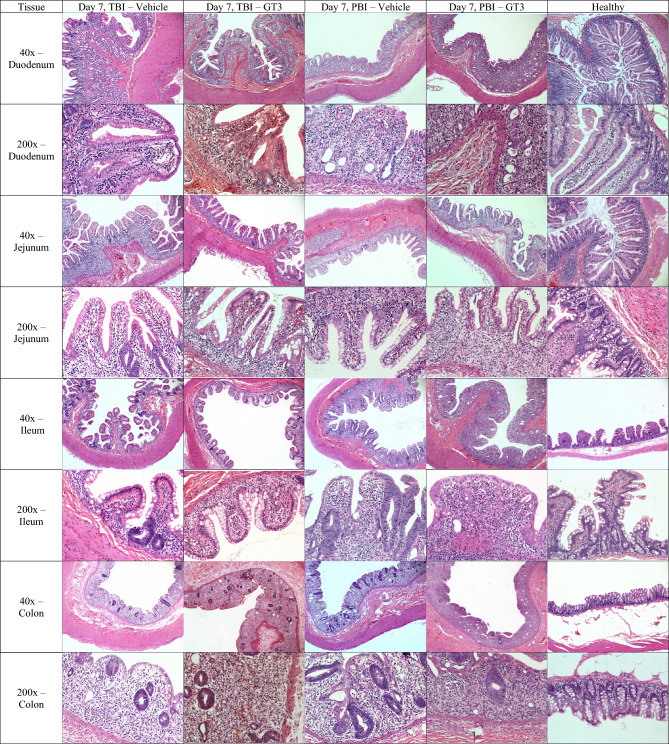
Comparison of gastrointestinal tissue sections collected from vehicle- and GT3-treated NHPs day 7 post-irradiation with 12 Gy (either partial-body or total-body irradiation (PBI/TBI)). GT3 Duodenum: TBI RA2528F, PBI 1603158F, Vehicle Duodenum: TBI RA3291F, PBI 1603103M, Healthy SD0F. GT3 Jejunum: TBI RA2829M, PBI 1603047 M, Vehicle Jejunum: TBI RA2378F, PBI 1605536F, Healthy SD0F. GT3 Ileum: TBI RA2528F, PBI 1603158F, Vehicle Ileum: TBI RA3291F, PBI 1603103M, Healthy SD0F. GT3 Colon: TBI RA2626F, PBI 1603158F, Vehicle Colon: TBI RA2378F, PBI 1605536F, Healthy SD0F.

The differences in the villi fusion and loss between the sections collected on day 4 for the two treatment groups (vehicle vs GT3-treated animals) were minimal in terms of the degree of villi fusion, degeneration and loss (Fig. 4, Supplementary Fig. 3, Table 6). By contrast, there was slightly greater loss and fusion of villi for the day 7 vehicle group compared to the GT3-treated group (Fig. 4, Supplementary Fig. 3, Table 6). The degree of villous loss and villi fusion was more severe for day 7 than the corresponding day 4 group, especially when the average values of each group were considered. Additionally, the villi to crypt ratios were consistently greater for tissues taken at day 4 than those values recorded at day 7 (Table 6).

##### Small intestine/jejunum

Samples of day 4 had a slightly greater loss and fusion and minimally greater inflammatory cellular infiltrates for the vehicle group compared to the GT3-treated NHPs, while no difference was noticed in the day 7 comparison (Table 6). No difference of average villous loss and fusion or the presence of inflammatory cellular infiltrates was noticed between vehicle and GT3 treatments on day 7. The crypt dilation was slightly more noticeable in the vehicle group compared to the GT3-treated counterpart. Distinct differences were observed between day 4 and day 7 animals, similar to the changes seen in the duodenum sections (Table 6). The degrees of villous loss and villi fusion were more severe for the day 7 animals than day 4 when the average values of each group were considered.

##### Small intestine/ileum

The histopathological changes of day 4 ileum sections exhibited similar changes and trends as those of the jejunum, with few exceptions. The ileum tissue sections of the vehicle-only group were characterized by mild villous loss and none to minimal villi fusion, while changes were slightly more evident in the GT3-treated group (mild to moderate villous loss and minimal to mild villi fusion) (Table 6). Crypt dilation was none to minimal dilation within the vehicle group compared to no dilation for the GT3-treated group. No clear difference in the inflammatory cell infiltrates was noticed between vehicle and GT3-treated counterparts for either day 4 and day 7 subsets (Table 6). Greater villous loss and fusion was seen in the GT3-treated animals versus the vehicle group collected on day 7. Similar results were observed on day 7; the vehicle group sections were characterized by mild to moderate villous loss and minimal to mild villi fusion, while the GT3-treated group presented with moderate to marked villous loss and minimal to moderate villi fusion. The vehicle group showed mild to moderate crypt dilation compared to the GT3-treated group with minimal to moderate dilation (Table 6).

##### Large intestine/colon

For the samples collected on day 4 and day 7, the vehicle group exhibited slightly greater crypt loss than the day 4 and day 7 GT3-treated NHPs, respectively (Fig. 4, Supplementary Fig. 3, Table 6). The degree of crypt loss appeared more severe within the day 7 sample group than seen within the earlier day 4 samples. No observable trend in degenerative crypt dilation or inflammatory cellular infiltrates was seen.

##### Liver

The tissue sections of the liver showed no discernable histopathological changes (relative to the tissues from unirradiated, control animals) following exposures to supra-lethal doses of TBI.

#### Urinary system- clinical features and histopathology

##### Clinical features

A few of the animals had dark urine output in their home cages at the time of euthanasia, which is a common sign of dehydration.

##### Histopathology/kidney

**Figure 5:**
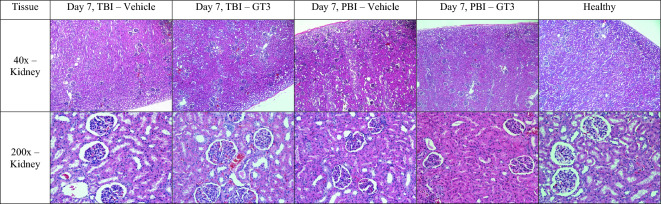
Comparison of kidneys collected from vehicle- and GT3-treated NHPs day 7 post-irradiation with 12 Gy. Radiation exposure was either partial-body (PBI) or total-body (TBI). GT3 Kidney: TBI RA2829M, PBI 1606085M, Vehicle Kidney: TBI RA2378F, PBI 1605536F, Healthy SD0F.

Kidneys of all supralethally irradiated animals under test showed comparable pathology in tubular degeneration, characterized by mild to marked dilated tubular lumen lined by attenuated tubular epithelial cells and lesser amounts of necrosis with variable loss of cytoplasm and dark, shrunken and round nuclei (Fig. 5, Supplementary Fig. 4, Table 6). The affected tubules were randomly distributed and mostly affected the cortex and to a lesser extent the medulla. Also present was tubular epithelial regeneration with large, round and slightly more basophilic nuclei that were arranged in close proximities (Fig. 5, Supplementary Fig. 4). Consistent patterns or degrees of tubular epithelial cell degeneration were not observed, nor were signs of epithelial regeneration within tissues of either the vehicle-treated or the GT3-treated animals (Fig. 5, Supplementary Fig. 4), despite the noted minor numerical changes in the scored pathology of kidney sections (Table 6). The tubular epithelial cell regeneration, however, was minimally greater, for the GT3-treated group compared to the vehicle-treated group. Moderate pathological changes were observed within the day 7 samples (Table 6).

##### Bladder

The tissue sections of the bladder demonstrated no discernable pattern(s) of histopathology (relative to the experimental time course and that of tissues from unirradiated controls) associated with the high dose (12 Gy) total-body exposure.

#### Cardiovascular system/clinical responses and histopathology

##### Clinical responses

The heart rates of all animals were within normal limits a few days post-irradiation. However, there were fluctuations in the overall heart rates that ranged from tachycardia to bradycardia. Towards the end of the study and at the time of euthanasia, all animals exhibited decreased heart rate.

##### Histopathology/heart

Tissue sections of the heart showed no definitive histopathology (i.e., relative to unirradiated control tissues) associated with the supralethal TBI.

#### Pulmonary system- clinical responses and/histopathology

##### Clinical responses

Overall, all of the animals showed a trend toward an increased rate of respiration a few days post-irradiation. The scheduled animals for euthanasia on day 4 exhibited fast to normal respiration rates. However, the breathing patterns transitioned to labored breathing by day 7 for the remaining animals.

##### Histopathology/lung

**Figure 6:**
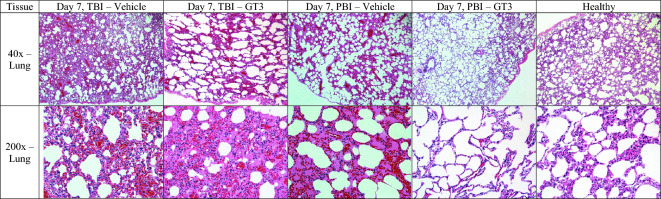
Comparison of lung tissue collected from vehicle- and GT3-treated NHPs day 7 post-irradiation with 12 Gy. Radiation exposure was either partial-body (PBI) or total-body (TBI). GT3 Lung: TBI RA2528F, PBI 1606085M, Vehicle Lung: TBI RA2922F, PBI 1605536F, Healthy 14041532F.

There were minimal differences in the extent of degenerative changes observed within day 4 lung samples (Fig. 6, Supplementary Fig. 5, Table 6). Mild to moderate alveolar degenerative changes were noted within lung tissues of vehicle- and GT3- treated animals. However, by day 7 post-exposure, the degree of lung pathology appeared to differ between vehicle- and GT3-treated NHPs; tissues of the vehicle-treated animals were characterized by moderate to marked alveolar septal degeneration, while tissues of the GT3-treated animals showed less pathologic mild to moderate changes (Table 6). There was a similar trend in the degree of edema within the alveoli of day 7 tissues. Distinct pathology was noted within lung tissues of the vehicle-treated animals: lesions were characterized by regionally marked edema composed of eosinophilic proteinaceous materials; septal structures were often diffused and regionally degenerated. Moderate septal degeneration with moderately thickened septa with surrounding mild to moderate hemorrhage that merged with eosinophilic proteinaceous material and expanded alveoli within the affected region was observed. The noted edema was likely the result of septal vascular compromise and septal structural degeneration. The GT3-treated animals showed multiple areas of moderate septal degeneration with moderately thickened septa with a corresponding partial loss of normal structure. There were surrounding areas with retained normal structures. In sum, a comparison between day 4 and day 7 tissues showed nearly one grade change in the extent of alveolar septal degeneration for both the vehicle- and GT3-treated animals. This reflects the slow sub-chronic nature of the progression of septal degeneration, given that the alveolar septum is composed of mature cells, i.e., alveolar epithelial cells (pneumocytes) and vascular endothelial cells that resist radiation damage more than mitotically active dividing cells. These sub-chronic degenerative histopathologic change progressions were observed from day 4 well into day 7.

### PBI-induced injuries in various organs

Similar to the TBI study, clinical and pathological responses of all animals under test were collected and assessed.

The primary clinical response metrics are briefly described with major findings listed; i.e., blood responses (Fig. 2), body weights (Table 1, Supplementary Table 2), body temperatures (Table 2, Supplementary Table 3), heart rates (Table 3, Supplementary Table 4), blood pressures (Table 4, Supplementary Table 5), gross pathology (Table 5) and histopathology (Table 6) of all irradiated animals receiving PBI.

#### Lymphohematopoietic system following PBI: clinical responses and histopathology

##### Clinical/blood responses

Blood responses of experimental animals receiving PBI (relative to TBI exposure animals) throughout the testing period are shown in Fig. 2. By comparison to TBI animals, the PBI animals maintained significantly higher WBC, lymphocyte, and neutrophil levels early during the post-exposure period (4 day and 7 day), while platelets were marginally higher, but not significantly so (Fig. 2). Of note, the GT3-treated PBI animals seemed to sustain higher RBC levels during the 4 day and 7 day post-exposure period (Fig. 2).

##### Histopathology/sternum

The sternum sections for the PBI cohort demonstrated a similar trend to that of the TBI animals which included severe hypoplasia, regardless of treatment (Fig. 3, Supplementary Fig. 2, Table 6).

##### Histopathology/spleen

There were little differences in the loss of splenic white pulp in the day 4 animals. In general, the day 7 spleen sections were characterized by diffused, marked to severe loss of white pulp cellularity and morphological losses of both the inner germinal zone and the outer mantle and peripheral zones. The spleen sections of the vehicle-treated subjects exhibited a range of pathologic changes such as marginal to marked loses in cellularity while those of GT3-treated animals tended to show slightly greater losses; however, these differences were minimal (Fig. 3, Supplementary Fig. 2, Table 6).

#### GI system following PBI- clinical responses and histopathology

##### Clinical/changes

Significant changes in body weights were observed in all NHPs subjected to supralethal PBI (or TBI), regardless of treatment (Table 1, Supplementary Table 2). At day 4, body weights of the GT3-treated animals under PBI were higher than those of vehicle-treated animals, which suggests that GT3 may have provided some form of clinical benefit to the animals. However, at the later time point of day 7, such differences in body weights were not obvious (Table 1, Supplementary Table 2). The majority of the animals showed significant dehydration (Table 5). Additionally, almost all of the animals exhibited decreased appetite and lethargy as the study progressed, all of which could have contributed to the significant body weight changes.

##### Histopathology/small intestine/duodenum

The differences in the villi fusion and loss between the day 4 subset were not clear under H&E stained slide evaluation. There may be slightly greater degenerative changes in the vehicle-group with little to sometimes marked changes noted when compared to the GT3-treated group that showed minimal to mild degenerative changes (Fig. 4, Supplementary Fig. 3). The villus/crypt ratio (V/C) ratio trends within the day 4 groups, relative to differences between the vehicle- and the GT3-treated animals were not obvious. Tissue sections from the day 7 NHPs regardless of treatment were characterized by moderate villous loss and mild to marked villi fusion (Table 6). The V/C ratio metric can be, and is often used, as an indicator of the degree of histopathology within the GI system (e.g., such as in cases of acute GI infections), although no such obvious trend was observed in this study.

Distinct differences were noticed between day 4 and day 7 groups. The degrees of villous loss and villi fusion were more severe for the day 7 animals than the corresponding day 4 animals (Table 6).

##### Histopathology/small intestine/jejunum

For NHPs euthanized on day 4, jejunum sections showed a different pattern than the day 7 counterparts (Table 6). However, there were minimal differences of villous loss and fusion and the number of inflammatory cellular infiltrates observed between the vehicle- and GT3-treated groups. For samples collected on day 7, lesser villous loss and fusion was seen for the vehicle-treated group than the GT3-treated group. This result suggested no beneficial effect of GT3 in this subset. Crypt dilation and the number of inflammatory cell infiltrates were slightly greater for the day 7 GT3-treated animals compared to day 7 vehicle-treated animals (Table 6).

Distinct differences were observed between day 4 and day 7 animals for the jejunum, similar to the changes seen in the duodenum (Fig. 4, Supplementary Fig. 3). Again, the degrees of villous loss and villi fusion were more severe for day 7 than day 4 when the average values of each group were considered. The V/C ratio was consistently greater for the day 4 jejunum sections compared to those for day 7.

##### Histopathology/small intestine/ileum

The histopathological changes of the ileum involved similar changes and trends as those of jejunum, with few exceptions. The day 4 vehicle sections were characterized by minimal to moderate villous loss and no to mild villi fusion, while the GT3-treated group displayed minimal to mild villous loss and no to minimal villi fusion. Equal crypt dilation was observed for both groups. The day 7 vehicle group was characterized by moderate to marked villous loss and mild to marked villi fusion. Changes were slightly more pronounced for the GT3-treated group, with moderate to marked villous loss and moderate to severe villi fusion. The crypt dilation data for the ileum sections contrasts those for the duodenum and jejunum tissue sections. The day 7 subsets show similar trends in slightly greater crypt dilation for the vehicle group compared to the GT3-treated group (Table 6).

No significant differences were noted between day 4 and day 7 tissues relative to the degree of inflammation (i.e., inflammatory infiltrates) within the ileum of vehicle- and GT3-treated animals. However, the V/C ratio was consistently greater for the ileum tissues for all day 4 groups compared to their day 7 counterparts.

##### Histopathology/large intestine/colon

Similar to the changes seen within the small intestine, distinct differences were noted between day 4 and day 7 groups for the large intestine. Both the day 4 vehicle- and GT3-treated groups showed similar degrees of crypt loss that ranged from mild to moderate. The day 7 vehicle group showed slightly less crypt loss than did the GT3-treated group (Table 6). Again, the degrees of crypt loss were more severe for day 7 than day 4 when the average values of each group were considered. The trend in the degenerative crypt dilation was not seen. Similarly, the differences in the inflammatory cellular infiltrates between various subgroups do not show consistent trends.

#### Additional organ systems evaluated following PBI: clinical responses and histopathology

##### Clinical responses/liver/urinary/cardiovascular systems

By a few days after irradiation, the heart rates of all animals were within normal limits, but changed around day 5 from irregular heart rates to slower heart rates at the time of euthanasia.

##### Histopathology/heart/liver/bladder/kidney

Similar to the TBI study, the tissue sections of heart, liver, and bladder of animals undergoing PBI showed no discernable histopathological patterns associated with exposure to supralethal doses of PBI.

#### Pulmonary system following PBI- clinical responses and histopathology:

##### Clinical/respiratory/pulmonary responses

In general, the respiration rates of the animals slowly increased after exposure to irradiation but decreased significantly by day 7. Some of the animals had difficulty breathing by the time of euthanasia.

##### Histopathology/pulmonary/lung

The difference in the extent of histopathological degenerative changes within lung samples collected on day 4 were minimal. There were generally minimal alveolar changes noted for the day 4 groups with the exception of the GT3-treated group, which demonstrated minimal to marked alveolar edema. By day 7, however, there were discernable differences between the GT3-treated and vehicle-treated NHPs. In the vehicle-treated group, greater alveolar septal degenerative changes were observed when compared with the GT3-treated group. The lung sections of the vehicle-treated group were characterized in general by minimal to mild alveolar septal degeneration, which was in contrast to a slightly broader range of minimal to moderate changes in the GT3 group. Alveolar septal degeneration and alveolar edema were slightly greater for the vehicle-treated group than the GT3-treated group. With the exception of one NHP, the lung sections of the GT3-treated group showed slightly milder histopathological changes as compared to the vehicle group (Table 6).

A comparison between day 4 and day 7 animals showed a similar trend as that of the TBI NHPs. A nearly one grade increase in alveolar septal degeneration was noted between the day 4 and day 7 samples.

## Discussion

This study was designed to explore and document the pathological consequences of supralethal exposures to IR using NHPs, and to evaluate the efficacy of a promising MCM, GT3, currently under development. Based on a few well-documented clinical cases involving accidentally exposed humans to supralethal doses of IR^52–59^, it is evident that an array of potentially lethal injuries often occurs within multiple organ systems of the body and that by simply correcting (via medicinal intervention) one organ system does not necessarily guarantee survival of the individual. Failure of multiple organ systems seems to dominate the landscape for selected individuals, thus tending to compromise the individual’s prospect of enjoying a long and healthy life^58,60,61^. As revealed by the histopathological evaluations of various tissues excised at necropsy following irradiation, the most severe and functionally compromising of these tissue-specific lesions observed were as follows: (A) *Lympho-hematopoietic tissues*, the sternum, showed marked cellular depletion/hypocellularity of active marrow, while in the spleen there were marked focal depletion of lymphoid-rich white-pulp; these IR-induced cellular deficits were reflected clearly by the noted depletion of circulating blood cells. (B) *GI tissues*, the three segments of the small intestine (duodenum/jejunum/ileum) all showed marked pathology of intestinal wall; the loss of villi, along with marked dilation of crypt, and the presence of inflammatory infiltrates all point to this pathology; whereas, within the large intestine (colon), there was comparable pathology with marked loss of crypt cells, increased crypt dilation and presence of inflammatory infiltrates within the intestinal wall. This GI-associated pathology was manifested clinically by rapid, dramatic decline in body weights of the heavily exposed animals, independent of the IR exposure regimen; (C) *Kidney* as a principal component of the urinary system exhibited pronounced pathology as evidenced by the tubular degenerative lesions and by the marginal compensatory regenerative responses of those vital structures, which was compounded by dehydration due to lack of water intake, a common symptom in irradiated animals; and finally, (D) The *lung*, as the primary component of the pulmonary system, exhibited several distinct types of pathological lesions early during the post-IR exposure period; the most prominent were degenerative alveolar/septal regions of the tissues, with increased cellularity of septal structures, along with edematous alveoli. In general, and as expected, histopathological changes were more severe in tissues collected on day 7 compared to tissues collected on day 4, which is representative of the time of progression of the degenerative processes for the various tissues analyzed.

By contrast to these striking pathological lesions found within select tissues/organ systems shortly after supralethal IR exposures, it was also surprising that none (i.e., distinctive pathological lesions) were noted within a number of other tissues examined; e.g., heart, liver, and bladder. No doubt, time was a critical factor at play here; i.e., the time required to express a given pathology within a given tissue/organ system differed and that for these tissues (i.e., heart, liver, bladder), that time to manifest these pathologies was simply too short (i.e., or conversely the latent period being too long) for these tissues. Recently, we reported that data gathered from both male and female NHPs under the same conditions provided different levels of clinical support over a range of acute, total-body gamma irradiation^62^. Under matched experimental conditions, we observed marginal, but evident differences between acutely irradiated male and female NHPs relative to the measured endpoints (survival rate, blood cell counts and cytokine concentrations). These differences appeared to be accentuated by the level of exposure as well as by the nature of clinical support. As presented in Fig. 1 which illustrates the experimental design, the number of males and females in each treatment group, exposure type, and day of sample collections are not enough to make conclusive sex comparisons.

Not only was our intent to examine the short-term pathological consequences of supralethal IR exposures within a tightly controlled, well-defined NHP model, we also wanted to examine the possibility that these high-dose IR injuries could be prevented or mitigated by applying a MCM, namely GT3, that has proven radioprotective efficacy, but at much lower IR exposure levels. Although our results were somewhat disappointing, they were not totally unexpected, as based on our prior experience with this MCM^31,34,40,43^. In recent studies, structural injuries and crypt survival were examined in the proximal jejunum of these supralethally irradiated animals on day 4 and 7 post-irradiation. Apoptotic cell death and crypt cell proliferation were assessed with TUNEL and Ki-67 immunostaining, and plasma citrulline was assessed using liquid chromatography-tandem mass spectrometry (LC–MS/MS). GT3 treatment improved these parameters to a limited extent^40,43^. Using lung and jejunum tissue from these supralethally irradiated NHPs, transcriptomic studies have also been conducted. Significant radiation-induced changes in the lung and jejunum transcriptome were demonstrated for TBI as well as PBI. However, no major influence of GT3 on radiation was noted in either comparison^44,45^.

It is well recognized that the radioprotective/radiomitigative actions of the vitamin E class of nutraceuticals to which GT3 belongs are generally confined to the lower intensities of IR that elicit less severe injuries, but tend to fail at significantly higher, more intense levels of exposure^31,32,34,40,43^. This attribute of IR dose-dependency (relative MCM efficacy) was in contrast to the aminothiol class of MCMs that clearly have a broader, more extensive range of radioprotectiveness^63^. The caveat is, however, that in contrast to the well-tolerated, essentially non-toxic GT3, aminothiols like amifostine are not well tolerated and are quite toxic when applied at efficacious doses^64,65^. Nevertheless, the results here and in prior studies hint that GT3 can indeed be selectively radioprotective; e.g., noting the sparing effect of single doses of GT3 has on vital intestinal crypt stem cells even under extreme high levels of IR exposure^40,43,66^. Can these protective actions of GT3 be improved upon and extended with changes in the treatment regimen or by combining with another agent? GT3 has been tested in combination with a few agents in the murine model with positive outcomes using radiation doses in the H-ARS range^38,67–69^. However, additional studies using higher radiation doses that will induce GI-ARS need to be accomplished. Time will tell as we fully intend to pursue answers to these questions.

## Materials and methods

### Experimental design and animals

This study was conducted for the primary purpose of evaluating a promising new MCM, GT3, that is currently under development. Secondarily, the study’s purpose was to detail the pathological consequences of supralethal doses (12 Gy) of IR (^60^Co gamma rays or linear accelerator generated high energy photons) delivered either as TBI or PBI in order to provide essential information for the safety and efficacy assessments of MCMs under test. Keeping in mind the 3Rs to minimize the use of animals, particularly NHPs, this pilot study was conducted with 8 animals in each group.

In brief, a total of 32 naïve rhesus macaques (*Macaca mulatta*, 18 females and 14 males) were used for this study. They weighed between 4.35 and 10.35 kg and were between 3.5 and 5.5 years of age. The NHPs were randomly assigned to a TBI or PBI cohort, 16 in each. Each group of 16 animals was further randomly divided; eight received GT3, and the remaining eight received vehicle. Four animals in each group were euthanized on day 4 and the remaining five on day 7 post-irradiation for tissue collection^44,45^. The plan was to euthanize 3 animals each on days 4 and 7, and the remaining 2 animals on day 10 post-irradiation in each group (vehicle and GT3). By day 7 post-irradiation, all animals were moribund requiring euthanasia to save them from pain and suffering. Thus, there are 3 animals for day 4, and 5 animals for day 7 post-irradiation. Unirradiated control animal samples (all rhesus: two females and one male, age 4–9 years, body weight 6.5–12 kg) obtained from commercial vendors were also used to assess comparatively the overall clinical and pathological effects of supralethal (a dose where there is hardly any expectation for survivors) exposures.

All animals were housed in a facility accredited by the Association for the Assessment and Accreditation of Laboratory Animal Care (AAALAC)-International. Additional animal details and their housing have been described earlier^34,70^. All animals under test were monitored clinically on a periodic basis throughout the testing period using standard veterinary/clinical practices^44,45^. These monitored clinical parameters included but were not limited to: gross behavioral changes, change in body weights, blood cell counts and differentials, patterns of defecation and urination, and heart rate and blood pressure changes.

All procedures involving animals were approved by the Institutional Animal Care and Use Committee (BIOQUAL Inc. Rockville, MD, USA, Protocol #18-060) and the Department of Defense Animal Care and Use Review Office (ACURO). The study was conducted in strict accordance with the recommendations in the *Guide for the Care and Use of Laboratory Animals*^71^. This study was carried out in compliance with the ARRIVE guideline.

### Drug preparation and administration

The MCM under evaluation was GT3. Both the MCM and its vehicle were procured from Callion Pharma (Jonesborough, TN, USA), and GT3 was supplied as an injectable suspension at a concentration of 50 mg/ml^34^. Either GT3 or the vehicle (olive oil 50 mg/ml) in 5% Tween-80 in saline were injected subcutaneously (*sc)* at a dose of 37.5 mg/kg 24 h prior to irradiation^47^. The individual weight of each NHP decided the volume of drug or vehicle administered.

### Irradiation

During any radiation exposure mass casualty scenario, victims will be exposed to either total-body or partial-body radiation (TBI/PBI). Thus, both types of radiation exposures are used for developing radiation countermeasures and studying radiation injuries. For PBI, we have spared 5% bone marrow (excluding the hind limbs, fibula, tibia, and feet). Due to such bone marrow sparing, PBI is less injurious than TBI. Recently, these results are published^42–45^. Animals were exposed to either cobalt-60 gamma-radiation (TBI) or LINAC-derived photon (PBI). We used two different radiation sources for PBI and TBI due to specific reasons. LINAC is preferred for PBI as it provides a collimated beam and the irradiation field is limited. With this method, it is possible to place only the desired part of the body under the irradiation field. The high-level cobalt-60 facility (HLCF) for gamma-radiation is used for TBI in our institution and has a panoramic field. It is not possible to provide complete sparing of any body part with this method. The radiation dose rate for TBI and PBI were 0.6 Gy/min and 1.3 Gy/min, respectively^34,43^. After irradiation, the NHPs were returned to their cages and were monitored for recovery from the irradiation procedure.

#### TBI

Based on the animals’ abdominal width, they were matched with another animal with a close measurement and subsequently irradiated as a pair. Each animal’s ID number and radiation dose were confirmed by the attending dosimetrist before proceeding with the procedure. During irradiation, they were constantly monitored via closed circuit TV monitors. The radiation field in the area of the NHP location was uniform within ± 1.5%. The dosimetry for photons was based on the alanine/EPR (electron paramagnetic resonance) dosimetry system^34,72^. This is one of the most precise dosimetry techniques at present, which is used by national standards laboratories for the most critical measurements and calibrations.

#### PBI

In brief, animals were fasted 12 h prior to the procedure. Once sedated, a single animal was transported to the LINAC facility where its ID number and radiation dose were verified by the dosimetrist. The animals’ heart rates and temperatures were monitored throughout the procedure. For PBI, 5% sparing of bone marrow was accomplished by excluding the hind limbs, fibula, tibia, and feet from the irradiation field. The Farmer ionization chamber used for dosimetry was calibrated at a National Institute of Standards and Technology-traceable accredited dosimetry calibration laboratory. The calibration was in terms of absorbed dose-to-water^73^. The absorbed dose rates determined from the ionization chamber measurements were used to determine a function of dose rate vs. phantom diameter (where phantom diameter corresponds to the anterior/posterior separation of the NHP).

For both TBI and PBI experiments, the study plan was to collect samples from 3 animals each on days 4 and 7 and two animals on day 10 post-irradiation. However, the animals scheduled for tissue collection on day 10 had to be euthanized on day 7 in accordance with the IACUC guidelines on moribundity.

### Blood and tissue collection

Blood was collected at various time points and analyzed as described earlier^62^. Tissue samples were collected immediately following euthanasia with EUTHASOL (Euthanasia solution, pentobarbital sodium and phenytoin sodium)^74^. Each animal was necropsied by a board-certified veterinarian experienced in performing gross anatomical pathological evaluations and any/all gross abnormalities were recorded. Tissue samples were placed into a histology cassette and transferred to a container filled with 10% buffered zinc formalin. Tissue samples collected included heart (apex), lung, sternum (bone marrow), peri-aorta, liver, spleen, kidney, and bladder. Tissues of the GI system (small intestine (duodenum, jejunum and ileum) as well as the large intestine (colon)) were excised from deeply anesthetized animals immediately prior to euthanasia, as approved in the institution’s IACUC protocol. We have archived all tissue samples for future use and tissue sharing with other investigators. Slides were then submitted to a veterinary pathologist for evaluation.

### Histopathology

Tissues from NHPs were fixed in 10% zinc-buffered formalin for paraffin embedding, sectioning, and hematoxylin and eosin (H&E) staining by Histoserv, Inc. (Germantown, MD, USA)^75^. Digital images of tissue sections were obtained with a Ziess Axioscan slide scanner and Zeiss Zen software (Carl Ziess Meditech, Inc., Dublin, CA, USA). An Olympus IX73 microscope (Olympus, Center Valley, PA) was used for 40 × magnification. Histological and morphometric analyses were performed by a Board-certified veterinary pathologist blinded to treatment groups. The severity of tissue damage was graded based on a point system, with 0 = none, 1 = minimal, 2 = mild, 3 = moderate, 4 = marked, and 5 = severe. A separate scale was used to interpret villi/crypt ratios: < 2 = severe, 2.1–3 = marked, 3.1–4 = moderate, 4.1–5 = mild, 5.1–6 = minimal, and > 6.1 = none.

### Statistical analysis

Statistical analysis of CBC and vital signs data was accomplished using statistical software IBM SPSS Statistics (version 28.0.1.1, https://www.ibm.com/products/spss-statistics). All error bars signify standard deviations. For CBC data, One-Way ANOVA tests were performed between GT3-treated groups and their respective vehicle groups (i.e., PBI GT3 vs. PBI Vehicle, and TBI GT3 vs. TBI Vehicle), vehicle-treated groups (i.e., PBI Vehicle vs TBI Vehicle), and GT3-treated groups (i.e., PBI GT3 vs. TBI GT3). Paired t-tests were also performed on CBC data to assess significant differences between the pre-exposure time points (days -7 and -3 combined) and day 4 and day 7, separately (Supplementary Table 1). For vital signs data, One-Way ANOVA tests were only performed between GT3-treated groups and their respective vehicle groups (i.e., PBI GT3 vs. PBI Vehicle, and TBI GT3 vs. TBI Vehicle). *P*-values < 0.05 were considered statistically significant for all tests performed.

### Ethics statement

All procedures were approved by the Institutional Animal Care and Use Committee AFRRI and BIOQUAL Inc., Rockville, and Department of Defense Animal Care and Use Review Office (ACURO). This study was carried out in strict accordance with the *Guide for the Care and Use of Laboratory Animals* of the National Institutes of Health.

### Supplementary Information

Supplementary Information.

## Data Availability

All relevant data are within the manuscript and supplementary material.
