# Supplementary material for: Histopathological studies of nonhuman primates exposed to supralethal doses of total- or partial-body radiation: influence of a medical countermeasure, gamma-tocotrienol
Source: Sci Rep. 2024 Mar 8;14:5757. doi: 10.1038/s41598-024-56135-w (PMC10923821; doi:10.1038/s41598-024-56135-w)
Supplement: Supplementary file 1 — Supplementary Information. [file 41598_2024_56135_MOESM1_ESM.docx]

**Supplementary Table 1.** Paired t-test results comparing CBC parameters at pre-irradiation time points (day -7 and -3 combined) to the post-irradiation time points, day 4 and day 7, separately

|  |  | Pre vs. day 4 | | Pre vs. day 7 | |
| --- | --- | --- | --- | --- | --- |
|  | Group | One-Sided | Two-Sided | One-Sided | Two-Sided |
| WBC | GT3 PBI | < .001 | < .001 | < .001 | < .001 |
|  | VEH PBI | < .001 | < .001 | < .001 | < .001 |
|  | GT3 TBI | < .001 | < .001 | < .001 | < .001 |
|  | VEH TBI | < .001 | < .001 | < .001 | .002 |
| RBC | GT3 PBI | .066 | .131 | .233 | .467 |
|  | VEH PBI | .003 | .006 | .224 | .448 |
|  | GT3 TBI | .004 | .008 | .056 | .112 |
|  | VEH TBI | <.001 | .001 | .106 | .211 |
| HCT % | GT3 PBI | .034 | .068 | .357 | .714 |
|  | VEH PBI | .001 | .002 | .179 | .358 |
|  | GT3 TBI | .003 | .006 | .002 | .005 |
|  | VEH TBI | <.001 | <.001 | .035 | .070 |
| PLT | GT3 PBI | .042 | .085 | <.001 | .001 |
|  | VEH PBI | .368 | .736 | .004 | .007 |
|  | GT3 TBI | .040 | .079 | <.001 | .001 |
|  | VEH TBI | <.001 | .001 | <.001 | <.001 |
| NEUT | GT3 PBI | <.001 | <.001 | .004 | .008 |
|  | VEH PBI | .002 | .003 | .003 | .005 |
|  | GT3 TBI | <.001 | <.001 | .001 | .003 |
|  | VEH TBI | <.001 | <.001 | .001 | .002 |
| LYMPH | GT3 PBI | <.001 | <.001 | <.001 | .001 |
|  | VEH PBI | <.001 | <.001 | <.001 | <.001 |
|  | GT3 TBI | <.001 | <.001 | <.001 | <.001 |
|  | VEH TBI | <.001 | <.001 | <.001 | .001 |

p-value of < 0.05 was considered significant by a one-sided and two-sided paired t-test.

**Supplementary Table 2.** Body weights of NHPs subjected to supralethal IR exposures (TBI or PBI) and treated with GT3 or vehicle

| Radiation | Treatment | Euthanasia (day) | NHP# | Sex | Age  (years) | % Weight Change (kg) | (N size), % change - Average ± SD | | |
| --- | --- | --- | --- | --- | --- | --- | --- | --- | --- |
|  |  |  |  |  |  |  | Total | Males | Females |
| PBI | Vehicle | 4 | 1608024 | F | 3.5 | -7 | (3) - 6.33 ± 0.58 | (1) - 6.00 | (2) - 6.50 ± 0.71 |
|  |  |  | 1603151 | M | 3.9 | -6 |  |  |  |
|  |  |  | 1603060 | F | 3.9 | -6 |  |  |  |
|  |  | 7 | 1607024 | F | 3.6 | -8 | (5) - 10.80 ± 1.92 | (3) - 11.3 ± 1.53 | (2) - 10.00 ± 2.80 |
|  |  |  | 1604091 | M | 3.8 | -13 |  |  |  |
|  |  |  | 1605536 | F | 3.8 | -12 |  |  |  |
|  |  |  | 1603103 | M | 3.9 | -10 |  |  |  |
|  |  |  | 1603155 | M | 3.9 | -11 |  |  |  |
|  | GT3 | 4 | 1603080 | F | 3.9 | -1 | (3) - 3.00 ± 2.65 | (1) - 6.00 | (2) - 1.50 ± 0.71 |
|  |  |  | 1608009 | F | 3.5 | -2 |  |  |  |
|  |  |  | 1603051 | M | 3.9 | -6 |  |  |  |
|  |  | 7 | 1606094 | F | 3.7 | -12 | (5) - 12.20 ± 2.28 | (3) - 11.00 ± 1.00 | (2) - 14.00 ± 2.83 |
|  |  |  | 1604041 | M | 3.8 | -11 |  |  |  |
|  |  |  | 1603158 | F | 3.9 | -16 |  |  |  |
|  |  |  | 1603047 | M | 3.9 | -10 |  |  |  |
|  |  |  | 1606085 | M | 3.7 | -12 |  |  |  |
| TBI | Vehicle | 4 | RA2619 | F | 5 | -6 | (3) - 6.33 ± 2.52 | (2) - 6.50 ± 3.53 | (1) - 6.00 |
|  |  |  | RA2692 | M | 4.9 | -4 |  |  |  |
|  |  |  | RA2892 | M | 3.7 | -9 |  |  |  |
|  |  | 7 | RA2378 | F | 5 | -14 | (5) - 10.8 ± 2.77 | (1) - 8.00 | (4) - 11.50 ± 2.65 |
|  |  |  | RA2931 | M | 4.2 | -8 |  |  |  |
|  |  |  | RA2922 | F | 4.2 | -11 |  |  |  |
|  |  |  | RA3238 | F | 3.8 | -13 |  |  |  |
|  |  |  | RA3291 | F | 3.9 | -8 |  |  |  |
|  | GT3 | 4 | RA2599 | M | 3.7 | 3 | (3) 0.67 ± 2.08 | (2) 1.00 ± 2.83 | (1) 0.00 |
|  |  |  | RA2781 | M | 5.1 | -1 |  |  |  |
|  |  |  | RA2115 | F | 5.5 | 0 |  |  |  |
|  |  | 7 | RA2829 | M | 5.2 | -8 | (5) - 12.20 ± 4.02 | (1) - 8.00 | (4) - 13.25 ± 3.77 |
|  |  |  | RA2626 | F | 5.2 | -9 |  |  |  |
|  |  |  | RA2528 | F | 4 | -18 |  |  |  |
|  |  |  | RA3246 | F | 3.9 | -14 |  |  |  |
|  |  |  | RA2762 | F | 5 | -12 |  |  |  |

±, standard deviation

**Supplementary Table 3.** Body temperatures of NHPs subjected to supralethal IR exposures (TBI or PBI) and treated with GT3 or vehicle

| Radiation | Treatment | Euthanasia (day) | NHP# | Sex | Age  (years) | Temperature (Celsius) recorded post-irradiation (day) | | | | | |
| --- | --- | --- | --- | --- | --- | --- | --- | --- | --- | --- | --- |
|  |  |  |  |  |  | -3 | Average ± SD  for day 3 | 4 | Average ± SD  for day 4 | 7 | Average ± SD  for day 7 |
| PBI | Vehicle | 4 | 1608024 | F | 3.5 | 39.67 | 39.50 ± 0.15 | 38.94 | 38.76 ± 0.32 |  |  |
|  |  |  | 1603151 | M | 3.9 | 39.44 |  | 38.94 |  |  |  |
|  |  |  | 1603060 | F | 3.9 | 39.39 |  | 38.39 |  |  |  |
|  |  | 7 | 1607024 | F | 3.6 | 39.67 | 39.72 ± 0.09 | 39.56 | 39.20 ± 0.67 | 37.72 | 36.66 ± 1.57 |
|  |  |  | 1604091 | M | 3.8 | 39.78 |  | 40.11 |  | 38.00 |  |
|  |  |  | 1605536 | F | 3.8 | 39.61 |  | 38.50 |  | 35.72 |  |
|  |  |  | 1603103 | M | 3.9 | 39.72 |  | 38.61 |  | 37.50 |  |
|  |  |  | 1603155 | M | 3.9 | 39.83 |  | 39.22 |  | 34.33 |  |
|  | GT3 | 4 | 1603080 | F | 3.9 | 38.67 | 38.04 ± 0.70 | 39.11 | 37.98 ± 1.30 |  |  |
|  |  |  | 1608009 | F | 3.5 | 38.17 |  | 38.28 |  |  |  |
|  |  |  | 1603051 | M | 3.9 | 37.28 |  | 36.56 |  |  |  |
|  |  | 7 | 1606094 | F | 3.7 | 39.44 | 39.54 ± 0.27 | 39.33 | 38.93 ± 0.68 | 34.78 | 35.43 ± 1.23 |
|  |  |  | 1604041 | M | 3.8 | 39.39 |  | 38.44 |  | 34.83 |  |
|  |  |  | 1603158 | F | 3.9 | 39.56 |  | 39.11 |  | 36.22 |  |
|  |  |  | 1603047 | M | 3.9 | 40.00 |  | 39.72 |  | 37.17 |  |
|  |  |  | 1606085 | M | 3.7 | 39.33 |  | 38.06 |  | 34.17 |  |
| TBI | Vehicle | 4 | RA2619 | F | 5.0 | 38.67 | 38.83 ± 0.44 | 38.22 | 38.22 ± 0.00 |  |  |
|  |  |  | RA2692 | M | 4.9 | 38.50 |  | 38.22 |  |  |  |
|  |  |  | RA2892 | M | 3.7 | 39.33 |  | 38.22 |  |  |  |
|  |  | 7 | RA2378 | F | 5.0 | 38.22 | 38.19 ± 0.43 | 38.44 | 38.58 ± 0.51 | 37.61 | 36.79 ± 0.77 |
|  |  |  | RA2931 | M | 4.2 | 38.33 |  | 38.72 |  | 36.44 |  |
|  |  |  | RA2922 | F | 4.2 | 37.44 |  | 38.06 |  | 35.83 |  |
|  |  |  | RA3238 | F | 3.8 | 38.50 |  | 39.39 |  | 36.50 |  |
|  |  |  | RA3291 | F | 3.9 | 38.44 |  | 38.28 |  | 37.56 |  |
|  | GT3 | 4 | RA2599 | M | 3.7 | 37.67 | 37.91 ± 1.27 | 38.00 | 38.48 ± 0.50 |  |  |
|  |  |  | RA2781 | M | 5.1 | 36.78 |  | 38.44 |  |  |  |
|  |  |  | RA2115 | F | 5.5 | 39.28 |  | 39.00 |  |  |  |
|  |  | 7 | RA2829 | M | 5.2 | 37.89 | 37.91 ± 0.40 | 38.11 | 38.27 ± 0.34 | 39.22 | 37.46 ± 1.75 |
|  |  |  | RA2626 | F | 5.2 | 37.67 |  | 38.44 |  | 39.28 |  |
|  |  |  | RA2528 | F | 4.0 | 38.50 |  | 38.33 |  | 36.17 |  |
|  |  |  | RA3246 | F | 3.9 | 37.44 |  | 37.78 |  | 35.44 |  |
|  |  |  | RA2762 | F | 5.0 | 38.06 |  | 38.67 |  | 37.17 |  |

±, standard deviation

**Supplementary Table 4.** Heart rates of NHPs subjected to supralethal IR exposures (TBI or PBI) and treated with GT3 or vehicle

| Radiation | Treatment | Euthanasia (day) | NHP# | Sex | Age  (years) | Heart Rate (BPM) recorded post-irradiation (day) | | | | | |
| --- | --- | --- | --- | --- | --- | --- | --- | --- | --- | --- | --- |
|  |  |  |  |  |  | -3 | Average ± SD for day -3 | Day 4 | Average ± SD for day 4 | Day 7 | Average ± SD for day 7 |
| PBI | Vehicle | 4 | 1608024 | F | 3.5 | 160 | 171 ± 22 | 210 | 210 ± 6 |  |  |
|  |  |  | 1603151 | M | 3.9 | 156 |  | 215 |  |  |  |
|  |  |  | 1603060 | F | 3.9 | 196 |  | 204 |  |  |  |
|  |  | 7 | 1607024 | F | 3.6 | 238 | 233 ± 22 | 212 | 225 ± 12 | 192 | 178 ± 33 |
|  |  |  | 1604091 | M | 3.8 | 269 |  | 223 |  | 200 |  |
|  |  |  | 1605536 | F | 3.8 | 224 |  | 224 |  | 184 |  |
|  |  |  | 1603103 | M | 3.9 | 216 |  | 224 |  | 196 |  |
|  |  |  | 1603155 | M | 3.9 | 220 |  | 244 |  | 120 |  |
|  | GT3 | 4 | 1603080 | F | 3.9 | 216 | 190 ± 45 | 188 | 208 ± 25 |  |  |
|  |  |  | 1608009 | F | 3.5 | 216 |  | 236 |  |  |  |
|  |  |  | 1603051 | M | 3.9 | 139 |  | 200 |  |  |  |
|  |  | 7 | 1606094 | F | 3.7 | 220 | 200 ± 19 | 220 | 213 ± 19 | 148 | 140 ± 27 |
|  |  |  | 1604041 | M | 3.8 | 180 |  | 200 |  | 172 |  |
|  |  |  | 1603158 | F | 3.9 | 180 |  | 200 |  | 116 |  |
|  |  |  | 1603047 | M | 3.9 | 216 |  | 244 |  | 108 |  |
|  |  |  | 1606085 | M | 3.7 | 204 |  | 200 |  | 156 |  |
| TBI | Vehicle | 4 | RA2619 | F | 5.0 | 248 | 249 ± 23 | 186 | 160 ± 24 |  |  |
|  |  |  | RA2692 | M | 4.9 | 227 |  | 153 |  |  |  |
|  |  |  | RA2892 | M | 3.7 | 273 |  | 140 |  |  |  |
|  |  | 7 | RA2378 | F | 5.0 | 250 | 236 ± 33 | 206 | 228 ± 25 | 158 | 173 ± 13 |
|  |  |  | RA2931 | M | 4.2 | 241 |  | 263 |  | 192 |  |
|  |  |  | RA2922 | F | 4.2 | 258 |  | 240 |  | 166 |  |
|  |  |  | RA3238 | F | 3.8 | 254 |  | 229 |  | 180 |  |
|  |  |  | RA3291 | F | 3.9 | 179 |  | 202 |  | 170 |  |
|  | GT3 | 4 | RA2599 | M | 3.7 | 121 | 159 ± 39 | 154 | 160 ± 7 |  |  |
|  |  |  | RA2781 | M | 5.1 | 157 |  | 167 |  |  |  |
|  |  |  | RA2115 | F | 5.5 | 198 |  | 160 |  |  |  |
|  |  | 7 | RA2829 | M | 5.2 | 137 | 202 ± 61 | 205 | 223 ± 27 | 158 | 187 ± 17 |
|  |  |  | RA2626 | F | 5.2 | 154 |  | 222 |  | 190 |  |
|  |  |  | RA2528 | F | 4.0 | 292 |  | 256 |  | 205 |  |
|  |  |  | RA3246 | F | 3.9 | 212 |  | 242 |  | 191 |  |
|  |  |  | RA2762 | F | 5.0 | 213 |  | 189 |  | 191 |  |

ND, not done; ±, standard deviation

**Supplementary Table 5.** Blood pressures of NHPs subjected to supralethal IR exposures (TBI or PBI) and treated with GT3 or vehicle

| Radiation | Treatment | Euthanasia (day) | NHP# | Sex | Age  (years) | Blood pressure recorded post-irradiation (day) | | | | | |
| --- | --- | --- | --- | --- | --- | --- | --- | --- | --- | --- | --- |
|  |  |  |  |  |  | -3 | Average ± SD for day -3 | Day 4 | Average ± SD for day 4 | Day7 | Average ± SD for day 7ay |
| PBI | Vehicle | 4 | 1608024 | F | 3.5 | 163/132 | 146/93 ± 24/36 | 148/84 | 126/80 ± 24/12 |  |  |
|  |  |  | 1603151 | M | 3.9 | 157/83 |  | 130/89 |  |  |  |
|  |  |  | 1603060 | F | 3.9 | 118/63 |  | 101/66 |  |  |  |
|  |  | 7 | 1607024 | F | 3.6 | 139/63 | 120/74 ± 24/20 | 135/93 | 129/88 ± 16/14 | 106/81 | 117/89 ± 21/18 |
|  |  |  | 1604091 | M | 3.8 | 82/56 |  | 111/78 |  | 107/88 |  |
|  |  |  | 1605536 | F | 3.8 | 121/87 |  | 139/76 |  | 130/108 |  |
|  |  |  | 1603103 | M | 3.9 | 117/63 |  | 146/111 |  | 148/105 |  |
|  |  |  | 1603155 | M | 3.9 | 143/105 |  | 112/83 |  | 96/65 |  |
|  | GT3 | 4 | 1603080 | F | 3.9 | 156/127 | 132/98 ± 27/26 | 106/77 | 124/76 ± 16/25 |  |  |
|  |  |  | 1608009 | F | 3.5 | 137/92 |  | 129/50 |  |  |  |
|  |  |  | 1603051 | M | 3.9 | 102/76 |  | 137/100 |  |  |  |
|  |  | 7 | 1606094 | F | 3.7 | 144/87 | 148/97 ± 11/10 | 124/67 | 121/77 ± 16/12 | 113/84 | 118/80 ± 16/20 |
|  |  |  | 1604041 | M | 3.8 | 160/104 |  | 142/91 |  | 96/65 |  |
|  |  |  | 1603158 | F | 3.9 | 149/108 |  | 108/71 |  | 123/82 |  |
|  |  |  | 1603047 | M | 3.9 | 132/87 |  | 103/66 |  | 117/60 |  |
|  |  |  | 1606085 | M | 3.7 | 155/100 |  | 126/90 |  | 139/111 |  |
| TBI | Vehicle | 4 | RA2619 | F | 5.0 | 149/82 | 160/93 ± 15/21 |  | 118/61 ± 10/24 |  |  |
|  |  |  | RA2692 | M | 4.9 | 177/80 |  |  |  |  |  |
|  |  |  | RA2892 | M | 3.7 | 154/117 |  |  |  |  |  |
|  |  | 7 | RA2378 | F | 5.0 | 158/99 | 168/98 ± 20/15 | 124/79 | 159/91 ± 20/29 | 135/87 | 116/78 ± 19/13 |
|  |  |  | RA2931 | M | 4.2 | 191/99 |  | 140/92 |  | 110/69 |  |
|  |  |  | RA2922 | F | 4.2 | 154/73 |  | 122/77 |  | 98/65 |  |
|  |  |  | RA3238 | F | 3.8 | 187/111 |  | 125/95 |  | 137/96 |  |
|  |  |  | RA3291 | F | 3.9 | 149/110 |  | 153/102 |  | 99/72 |  |
|  | GT3 | 4 | RA2599 | M | 3.7 | 110/63 | 136/73 ± 24/12 |  | 102/59 ± 6/9 |  |  |
|  |  |  | RA2781 | M | 5.1 | 139/87 |  |  |  |  |  |
|  |  |  | RA2115 | F | 5.5 | 158/69 |  |  |  |  |  |
|  |  | 7 | RA2829 | M | 5.2 | 89/48 | 137/83 ± 36/25 | 96/79 | 153/93 ± 11/23 | 117/85 | 128/89 ± 31/26 |
|  |  |  | RA2626 | F | 5.2 | 125/66 |  | 92/55 |  | 182/130 |  |
|  |  |  | RA2528 | F | 4.0 | 154/94 |  | 145/88 |  | 118/69 |  |
|  |  |  | RA3246 | F | 3.9 | 134/103 |  | 127/75 |  | 100/67 |  |
|  |  |  | RA2762 | F | 5.0 | 185/103 |  | 169/124 |  | 125/95 |  |


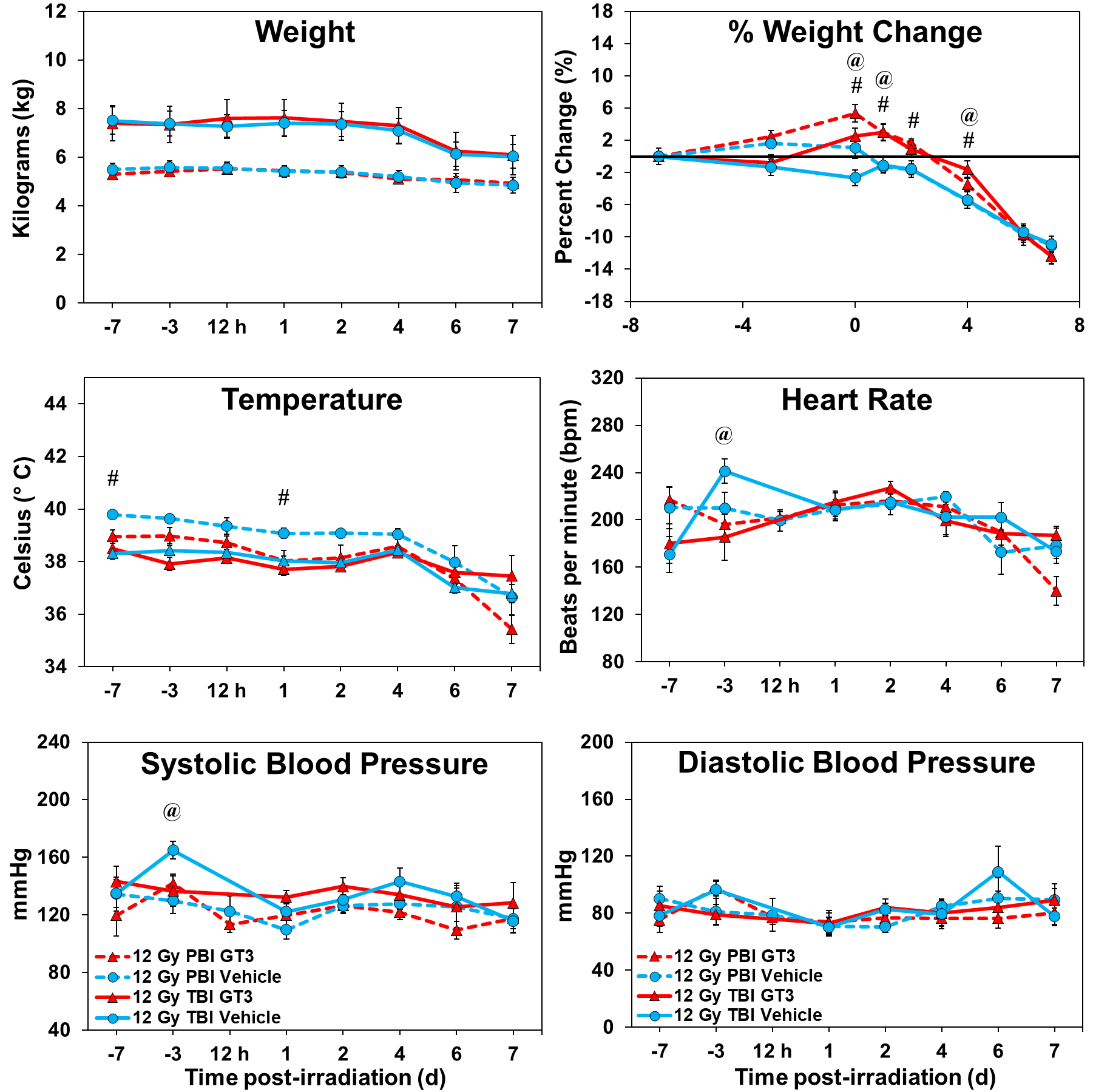


**Supplementary Figure 1.** Weight, percent weight change, temperature, heart rate, and systolic and diastolic blood pressure changes in NHPs treated with GT3 or vehicle and exposed to 12.0 Gy partial-body or total-body radiation. One-Way ANOVA tests were performed to compare differences between the GT3-treated groups and their respective vehicle-treated groups. Statistically significant changes between GT3-treated and vehicle-treated animals exposed to partial-body radiation are indicated by #, while significant changes between GT3-treated and vehicle-treated animals exposed to total-body radiation are indicated by @ (*p*-value < .05).

| Tissue | Day 4, TBI – Vehicle | Day 4, TBI – GT3 | Day 4, PBI – Vehicle | Day 4, PBI – GT3 |
| --- | --- | --- | --- | --- |
| 40x – Sternum | 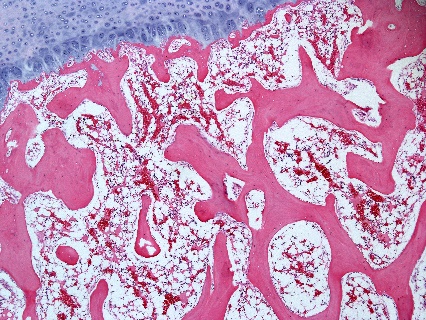 | 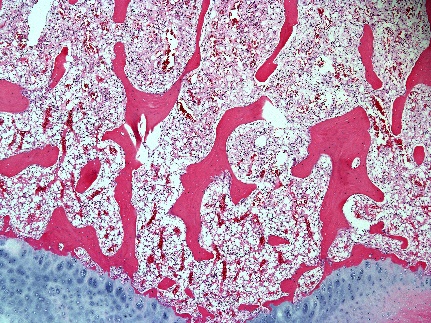 | 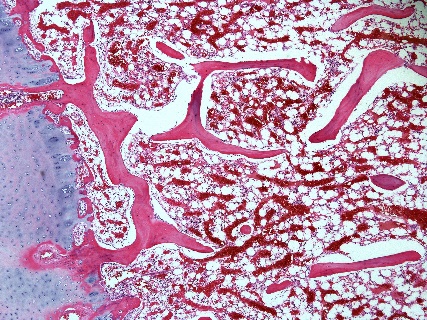 | 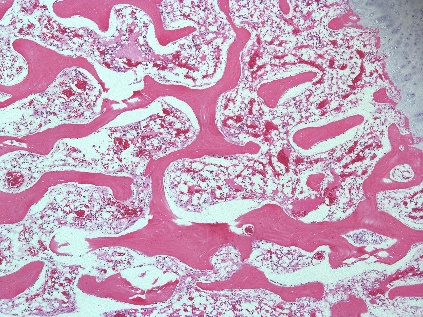 |
| 200x – Sternum | 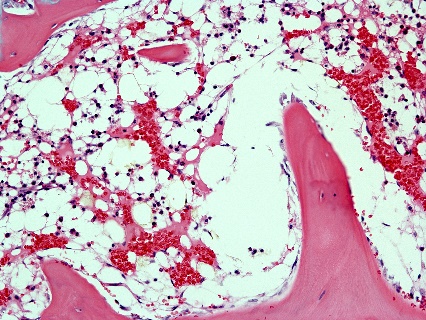 | 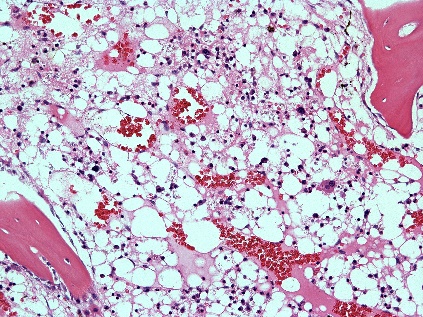 | 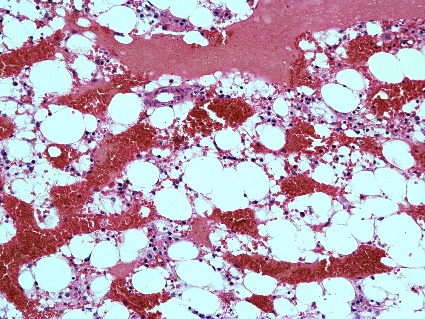 | 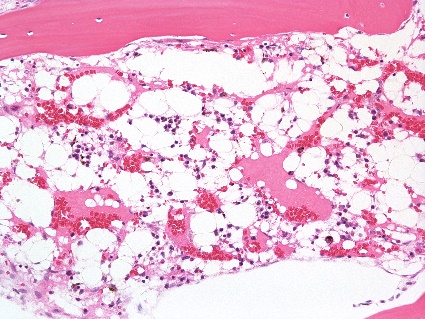 |
| 40x –  Spleen | 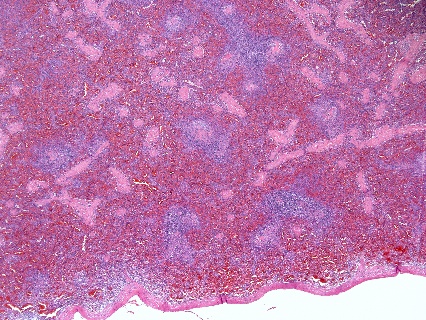 | 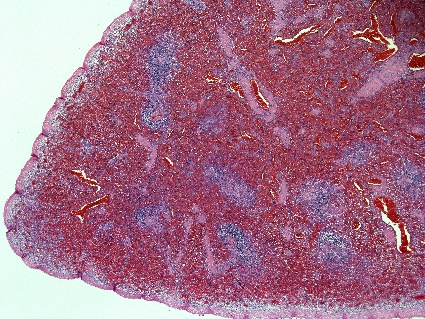 | 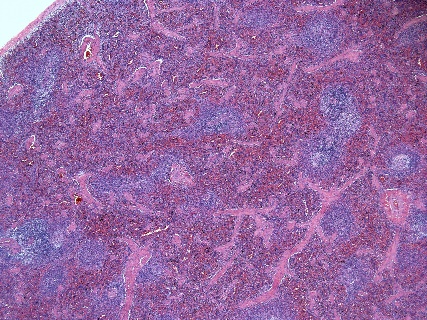 | 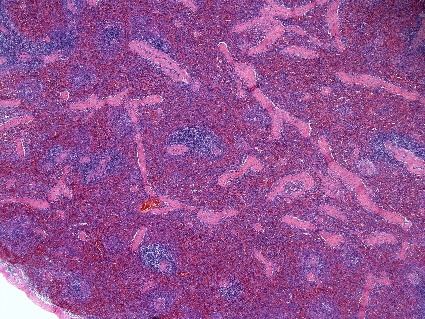 |
| 200x – Spleen | 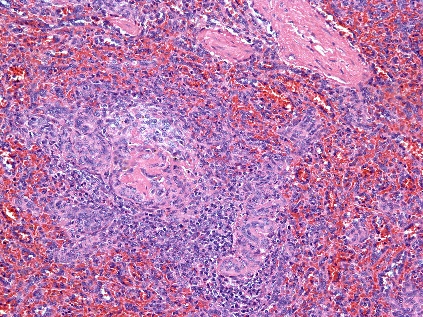 | 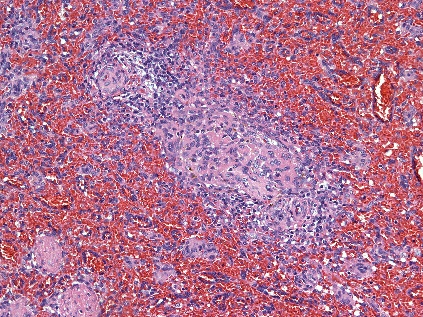 | 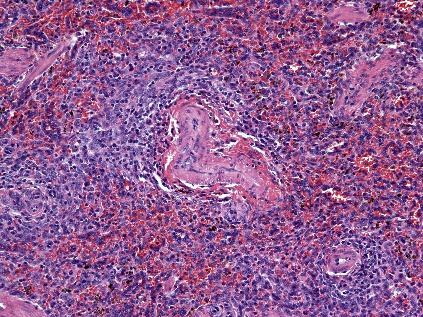 | 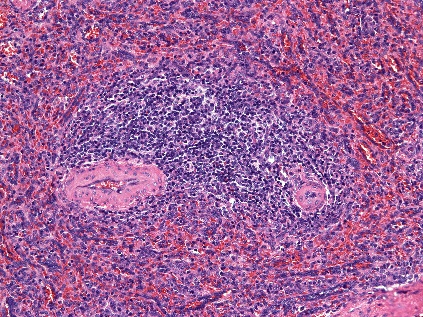 |

**Supplementary Figure 2.** Comparison of lymphohematopoietic organs collected from vehicle- and GT3-treated NHPs day 4 post-irradiation with 12 Gy. Radiation exposure was either partial-body (PBI) or total-body (TBI). Sternum: Vehicle TBI RA2619F, GT3 TBI RA2115F, Vehicle PBI 1603060F, GT3 PBI 1603080F. Spleen: Vehicle TBI RA2692M, GT3 TBI RA2781M, Vehicle PBI 1603151M, GT3 PBI 1603051M.

| Tissue | Day 4, TBI – Vehicle | Day 4, TBI – GT3 | Day 4, PBI – Vehicle | Day 4, PBI – GT3 |
| --- | --- | --- | --- | --- |
| 40x – Duodenum | 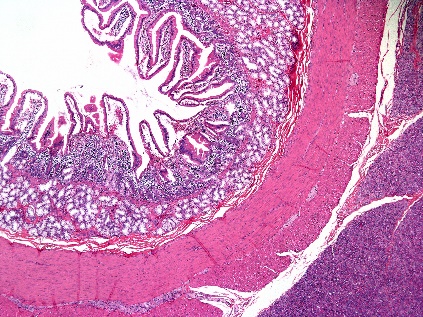 | 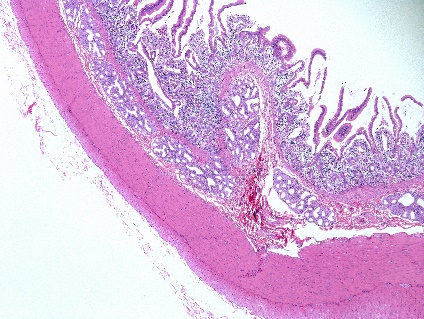 | 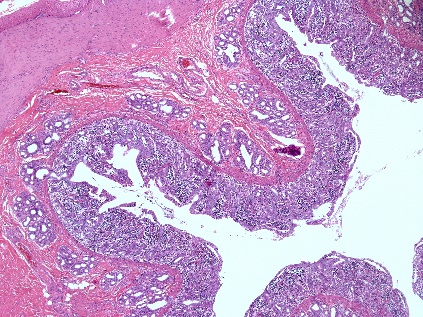 | 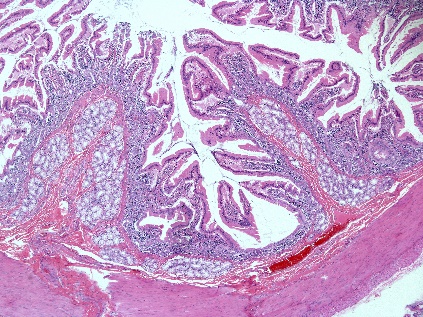 |
| 200x – Duodenum | 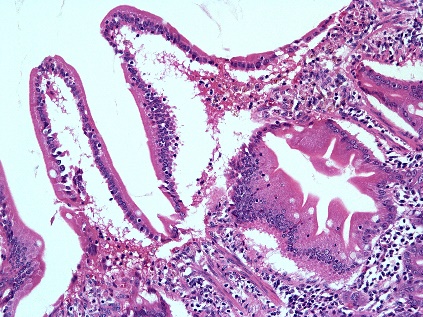 | 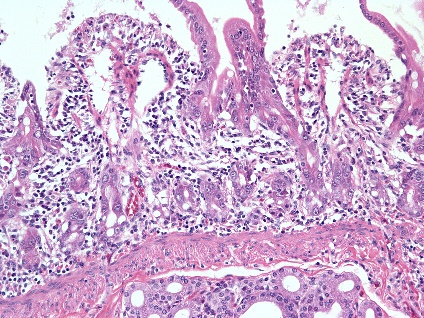 | 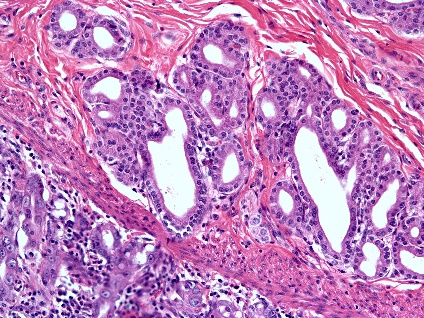 | 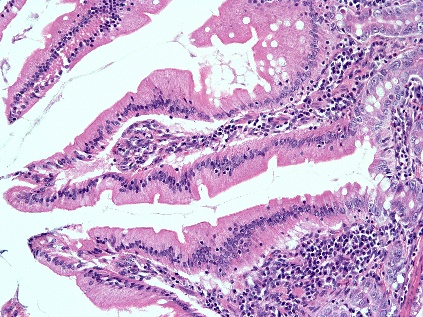 |
| 40x – Jejunum | 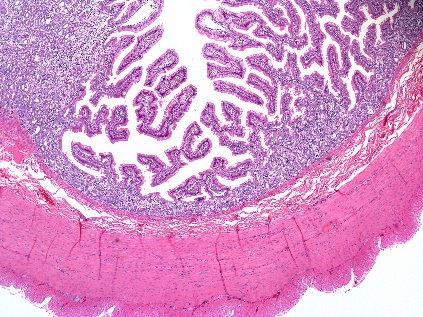 | 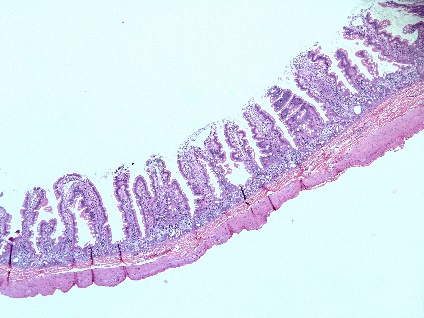 | 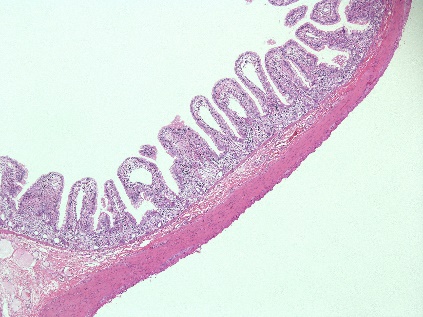 | 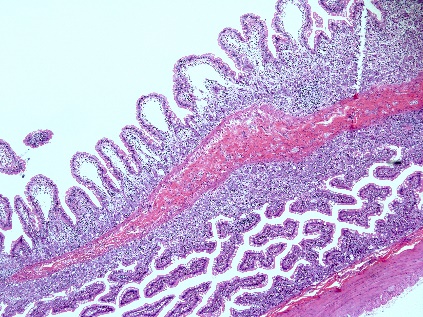 |
| 200x – Jejunum | 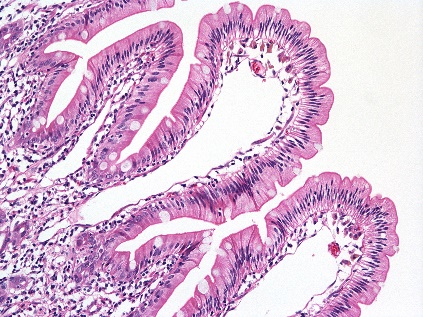 | 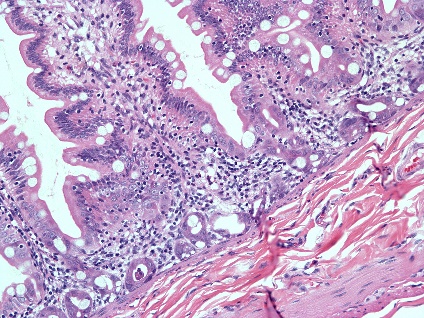 | 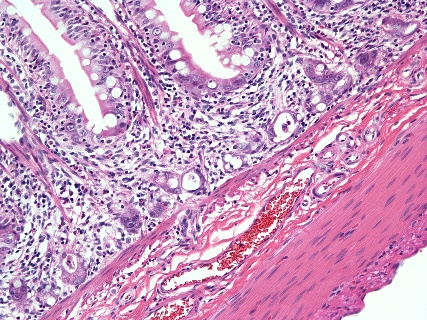 | 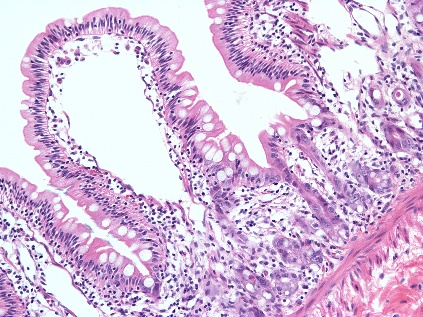 |
| 40x –  Ileum | 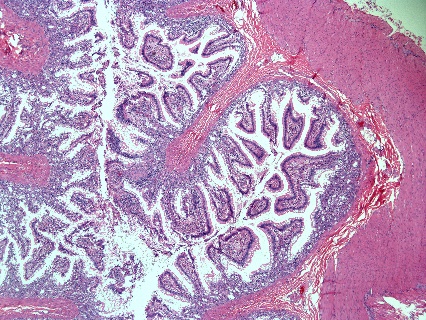 | 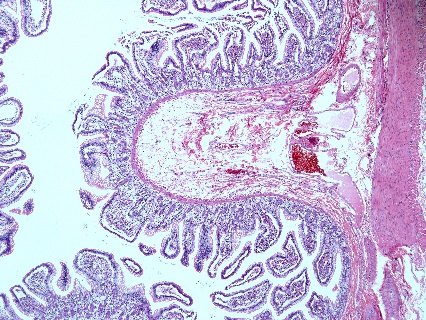 | 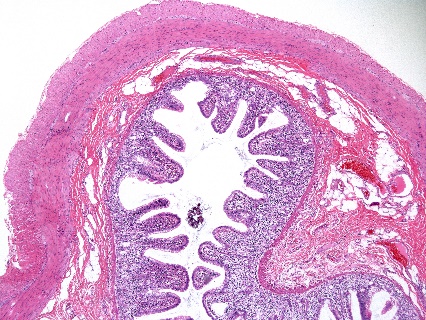 | 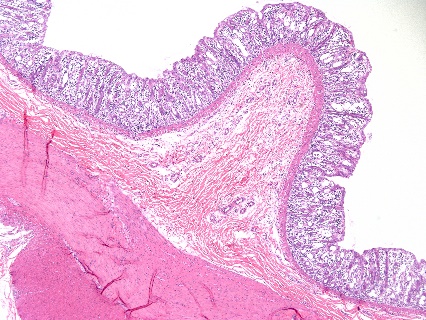 |
| 200x –  Ileum | 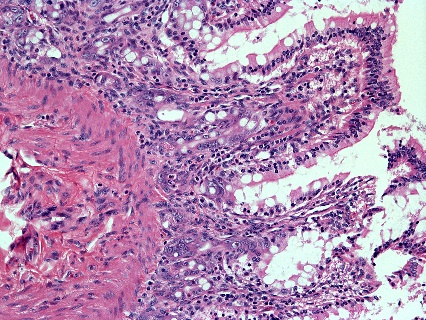 | 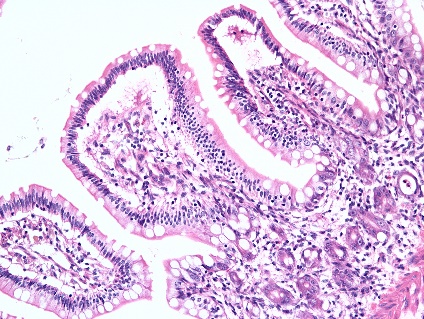 | 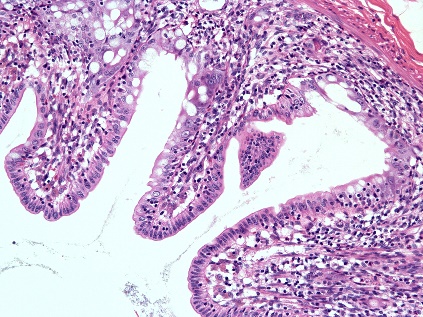 | 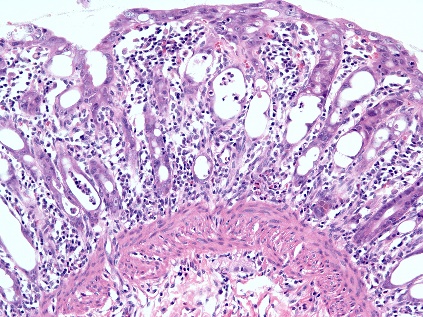 |
| 40x –  Colon | 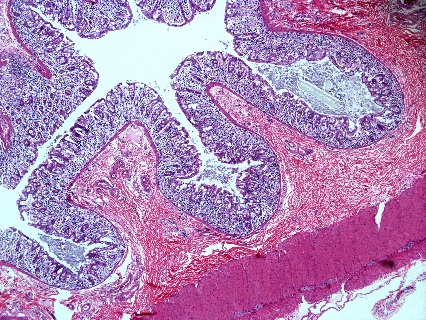 | 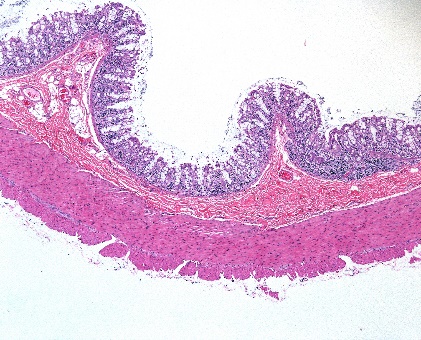 | 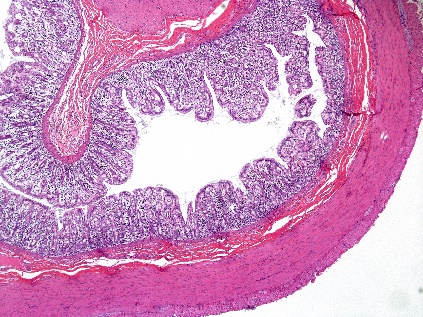 | 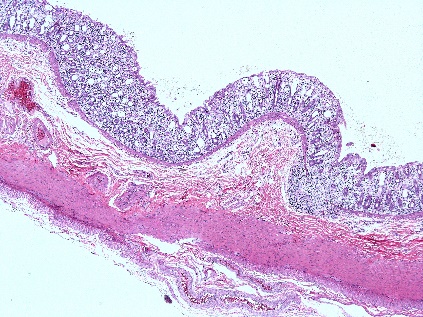 |
| 200x –  Colon | 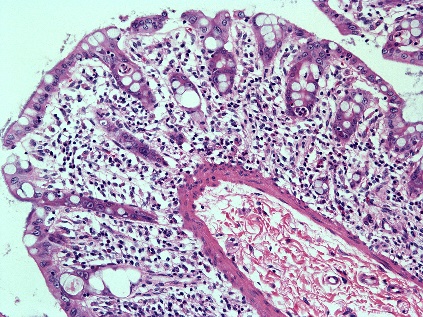 | 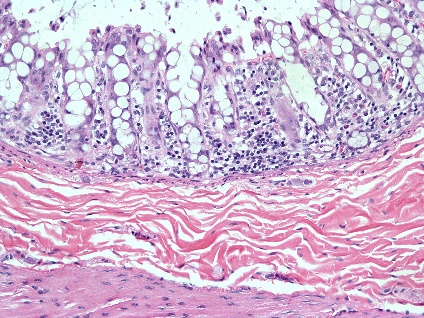 | 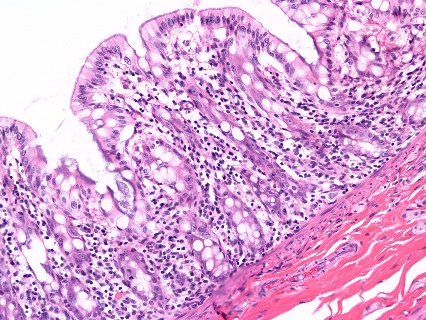 | 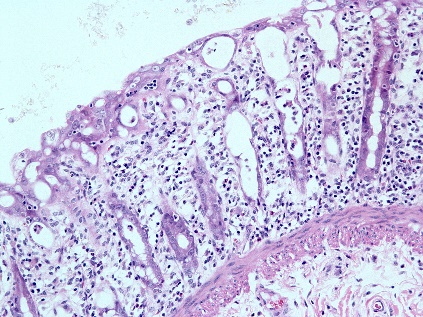 |

**Supplementary Figure 3.** Comparison of gastrointestinal tissue sections collected from vehicle- and GT3-treated NHPs day 4 post-irradiation with 12 Gy (either partial-body or total-body irradiation (PBI/TBI)). Duodenum: Vehicle TBI RA2892M, GT3 TBI RA2115F, Vehicle PBI 1603151M, GT3 PBI 1603051M. Jejunum: Vehicle TBI RA2692M, GT3 TBI RA2599M, Vehicle PBI 1603151M, GT3 PBI 1603051M. Ileum: Vehicle TBI RA2692M, GT3 TBI RA2115F, Vehicle PBI 1603151M, GT3 PBI 1603051M. Colon: Vehicle TBI RA2892M, GT3 TBI RA2599M, Vehicle PBI 1608024F, GT3 PBI 1608009F.

| Tissue | Day 4, TBI – Vehicle | Day 4, TBI – GT3 | Day 4, PBI – Vehicle | Day 4, PBI – GT3 |
| --- | --- | --- | --- | --- |
| 40x – Kidney | 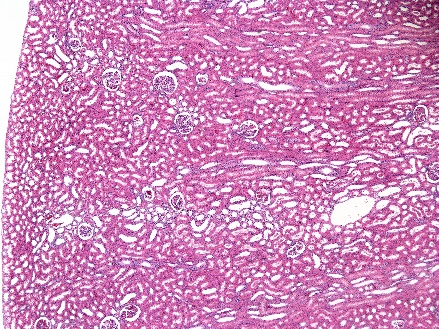 | 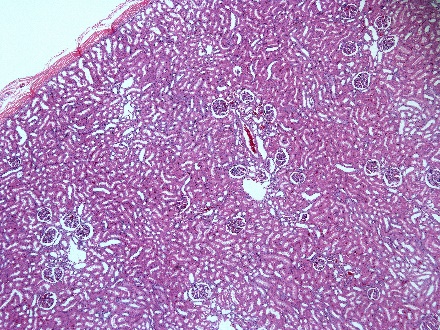 | 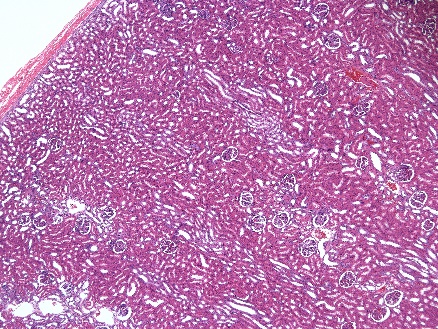 | 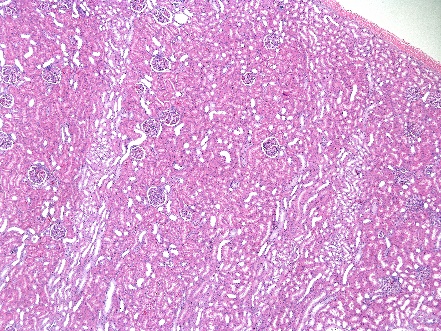 |
| 200x – Kidney | 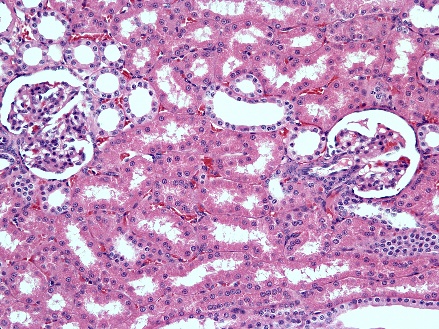 | 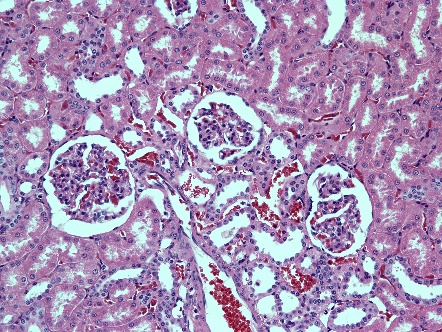 | 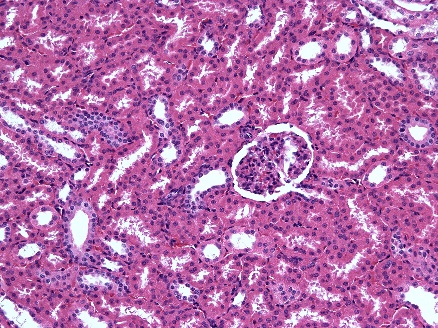 | 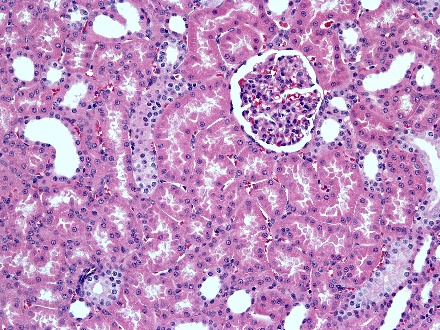 |

**Supplementary Figure 4.** Comparison of kidneys collected from vehicle- and GT3-treated NHPs day 4 post-irradiation with 12 Gy. Radiation exposure was either partial-body (PBI) or total-body (TBI). Kidney: Vehicle TBI RA2892M, GT3 TBI RA2599M, Vehicle PBI 1608024F, GT3 PBI 1608009F.

| Tissue | Day 4, TBI – Vehicle | Day 4, TBI – GT3 | Day 4, PBI – Vehicle | Day 4, PBI – GT3 |
| --- | --- | --- | --- | --- |
| 40x –  Lung | 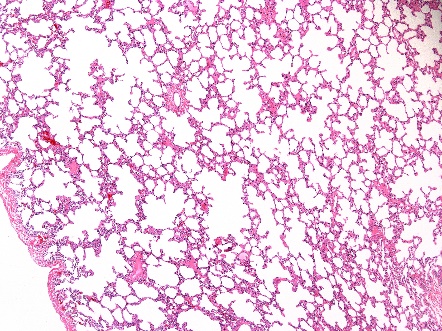 | 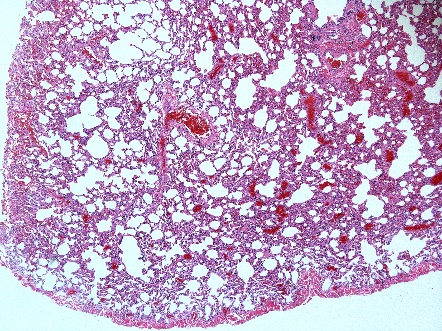 | 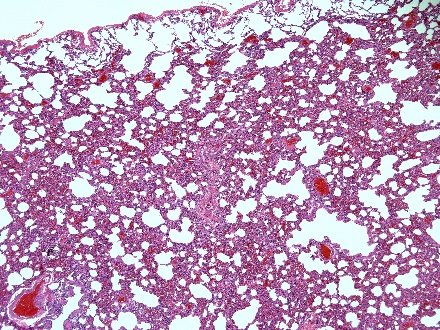 | 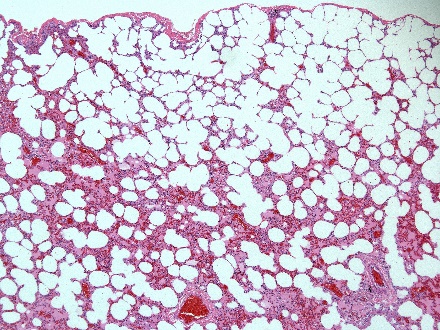 |
| 200x –  Lung | 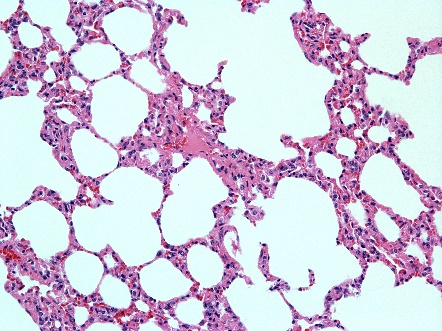 | 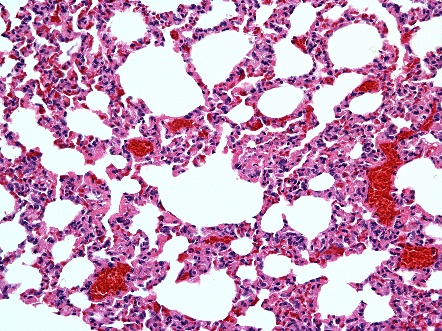 | 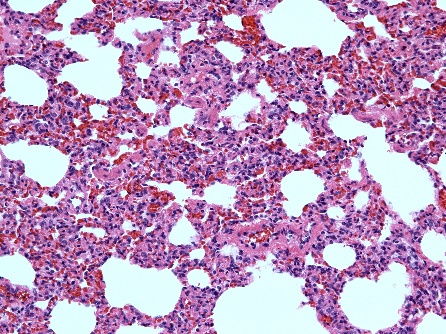 | 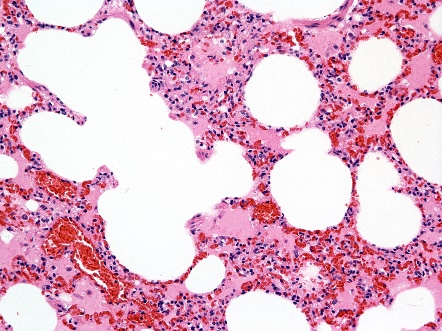 |

**Supplementary Figure 5.** Comparison of lung tissue collected from vehicle- and GT3-treated NHPs day 4 post-irradiation with 12 Gy. Radiation exposure was either partial-body (PBI) or total-body (TBI). Lung: Vehicle TBI RA2692M, GT3 TBI RA2781M, Vehicle PBI 1608024F, GT3 PBI 1603080F.
